# Nitrosative Redox Homeostasis and Antioxidant Response Defense in Disused *Vastus lateralis* Muscle in Long-Term Bedrest (Toulouse Cocktail Study)

**DOI:** 10.3390/antiox10030378

**Published:** 2021-03-03

**Authors:** Dieter Blottner, Daniele Capitanio, Gabor Trautmann, Sandra Furlan, Guido Gambara, Manuela Moriggi, Katharina Block, Pietro Barbacini, Enrica Torretta, Guillaume Py, Angèle Chopard, Imre Vida, Pompeo Volpe, Cecilia Gelfi, Michele Salanova

**Affiliations:** 1 Institute of Integrative Neuroanatomy, Charité—Universitätsmedizin Berlin, Corporate Member of Freie Universität Berlin, Humboldt-Universität zu Berlin, and Berlin Institute of Health, 10115 Berlin, Germany; dieter.blottner@charite.de (D.B.); gabor.trautmann@charite.de (G.T.); imre.vida@charite.de (I.V.); 2 Center of Space Medicine Berlin, 10115 Berlin, Germany; guidogambara@gmail.com (G.G.); katharina.block@charite.de (K.B.); 3 Department of Biomedical Sciences for Health, University of Milan, Via Luigi Mangiagalli 31, 20133 Milan, Italy; daniele.capitanio@unimi.it (D.C.); Manuela.Moriggi@grupposandonato.it (M.M.); pietro.barbacini@unimi.it (P.B.); cecilia.gelfi@unimi.it (C.G.); 4 C.N.R. Institute of Neuroscience, 35121 Padova, Italy; sfurlan@mail.bio.unipd.it; 5 IRCCS Policlinico S. Donato, Piazza Edmondo Malan 2, 20097 San Donato Milanese, Italy; 6 IRCCS Istituto Ortopedico Galeazzi, Via Riccardo Galeazzi 4, 20161 Milan, Italy; enrica.torretta@grupposandonato.it; 7 UFR STAPS, INRAE, Université de Montpellier, UMR 866 Dynamique et Métabolisme, 34060 Montpellier, France; guillaume.py@umontpellier.fr (G.P.); angele.chopard@umontpellier.fr (A.C.); 8 Department of Biomedical Sciences, University of Padova, 35122 Padova, Italy; pompeo.volpe@unipd.it

**Keywords:** oxidative stress, skeletal muscle redox homeostasis, antioxidant systems, RNS in cell signaling, bedrest muscle disuse, sarcopenia

## Abstract

Increased oxidative stress by reactive oxygen species (ROS) and reactive nitrogen species (RNS) is a major determinant of disuse-induced muscle atrophy. Muscle biopsies (thigh vastus lateralis, *VL*) obtained from healthy male subjects enrolled in the Toulouse Cocktail bedrest (BR) study were used to assess efficacy of an antioxidant cocktail (polyphenols, omega-3, vitamin E, and selenium) to counteract the increased redox homeostasis and enhance the antioxidant defense response by using label-free LC–MS/MS and NITRO-DIGE (nitrosated proteins), qPCR, and laser confocal microscopy. Label-free LC–MS/MS indicated that treatment prevented the redox homeostasis dysregulation and promoted structural remodeling (TPM3, MYH7, MYBPC, MYH1, MYL1, HRC, and LUM), increment of RyR1, myogenesis (CSRP3), and skeletal muscle development (MUSTN1, LMNA, AHNAK). These changes were absent in the Placebo group. Glycolysis, tricarboxylic acid cycle (TCA), oxidative phosphorylation, fatty acid beta-oxidation, and mitochondrial transmembrane transport were normalized in treated subjects. Proteins involved in protein folding were also normalized, whereas protein entailed in ion homeostasis decreased. NITRO-DIGE analysis showed significant protein nitrosylation changes for CAT, CA3, SDHA, and VDAC2 in Treatment vs. Placebo. Similarly, the nuclear factor erythroid 2-related factor 2 (Nrf-2) antioxidant response element (Nrf-2 ARE) signaling pathway showed an enhanced response in the Treatment group. Increased nitrosative redox homeostasis and decreased antioxidant defense response were found in post-BR control (Placebo, *n* = 10) vs. the antioxidant cocktail treated group (Treatment, *n* = 10). Taken together, increased nitrosative redox homeostasis and muscle deterioration during BR-driven physical inactivity were prevented, whereas decreased antioxidant nitrosative stress defense response was attenuated by Treatment suggesting positive effects of the nutritional intervention protocol in bedrest.

## 1. Introduction

A large body of evidence indicates that after extended periods of reduced physical activity, occurring in many clinical settings, in musculoskeletal disorders and during long-term bedrest immobilization or spaceflights, muscles (especially those of the lower limb) undergo metabolic structural and functional adaptations resulting in skeletal muscle atrophy [1]. Skeletal muscle deconditioning is due to altered muscle proteostasis caused by imbalance between protein synthesis, which decreases, and protein degradation, which increases under reduced physical activity [2]. Several studies, however, have proposed that increased production of reactive oxygen species (ROS) and reactive nitrogen species (RNS) in skeletal muscle plays a synergistic role as signaling mechanism contributing to disuse-induced muscle atrophy by enhancing protease activity and repressing protein synthesis [3,4].

In bedrest-induced muscle disuse (BR), the level of nitrosylated muscle proteins involved in the control of Ca^2+^ homeostasis is significantly increased [5]. Increased autoregulatory *S*-nitrosylation of nitric oxide synthase type-1 (NOS1) has been observed after 60 days of bedrest [5], i.e., it is a promoter of functional *S*-nitrosylated (SNO) protein increase [5]. In BR, some of these changes were significantly reduced by resistive vibration exercise (RVE), and to lesser extent by resistive exercise (RE) countermeasure, indicating the presence of variable exercise response mechanisms that triggers redox homeostasis in human muscle [5,6]. In the mdx mouse model, the age-dependent RyR1 nitrosylation leads to channel remodeling resulting in “leaky” channels [7] with increased intracellular calcium release and increased calcium-dependent calpain protease activity [8].

In this context, antioxidant treatments via nutrient supplementation in animal models have been proposed as a countermeasure to protect muscles against disuse-induced atrophy [9]. Vitamin E treatment turned out to share antioxidant capacities by acting as a scavenger of reactive oxygen species (ROS), and as anti-inflammatory agent, through the inhibition of the NF-kB signaling pathway [10,11]. Moreover, vitamin E supplementation mitigates disuse atrophy in rats [12] and in obese subjects, reducing adipose tissue fibrosis, inflammation, oxidative stress, and lipids levels [13]. In the elderly, vitamin E supplementation was found to be associated with muscle strength improvement [14].

Vitamin E in combination with selenium as ROS scavenger displayed therapeutic effects in dystrophic muscle [15]. Additionally, selenium deficiency characterizes several skeletal muscle disorders [16]; thus, supplementation of selenium and selenoproteins have been adopted in prevention of muscle atrophy [17]. Studies in healthy human subjects, in patients, and in animal models suggested that omega-3 fatty acid treatment influences muscle metabolism and lipidogenesis [18] with therapeutic effects on inflammation and oxidative stress [19]. Of note, omega-3 supplementation during bedrest and spaceflight showed protective effects on unloaded muscles [20].

The present study stems from recent observations [21,22,23] demonstrating the additive and synergistic effects of a combined use of substances such as polyphenols, vitamins, and essential fatty acids to mitigate some metabolic features induced by extended physical inactivity [24].

We report on findings from the 60 days Toulouse Cocktail (2017–2018) bedrest study with healthy male participants. In this study “exercise” as countermeasure was replaced by “antioxidant substances” as treatment consisting of a cocktail composed of polyphenols, omega-3, vitamin E, and selenium to test their efficacy in preventing increased redox activity under extended disuse conditions. Results indicate that antioxidants supplementation during bed rest can mitigate muscle protein dysregulation promoting proteostasis, protecting from nitrosative stress and enhancing antioxidative defense response.

Biopsy samples were analyzed at the tissue level in the Treatment vs. Placebo group by label-free LC–MS/MS (proteomics), NITRO-DIGE (protein *S*-nitrosation), qPCR, and high-resolution laser confocal microscopy to investigate (i) proteomic changes, (ii) nitrosation levels of muscle proteins, (iii) transcription of antioxidant defense enzymes from the family of antioxidant responsive elements (ARE genes), and (iv) Nrf2 transcription and myonuclear protein accumulation.

## 2. Materials and Methods

### 2.1. Subjects and Ethics Statement

Twenty healthy male subjects were selected in 2 different 60-day 6° head-down tilt (HDT) bed rest (BR) campaigns (age: 34 ± 7.8; height: 176 ± 5 cm; weight: 73.5 ± 6.1 kg; body mass index (BMI): 23.7 ± 1.5. Medical selection criteria were restricted to no history or physical signs of neuromuscular disorders, non-smokers, and not taking drugs or medications.

The study was sponsored by the ESA (European Space Agency) and conducted at the Space Clinic of the Institute of Space Medicine and Physiology (Medes-IMPS, Rangueil Hospital) in Toulouse (France) according to the guidelines of the Declaration of Helsinki, and approved by the Ethics Committee CPP Sud-Ouest et Outre-Mer I, France (number ID RCB: 2016-A00401–50). A more detailed experiment protocol is reported elsewhere [25].

Altogether, 20 male subjects were recruited for the study that were conducted in two different campaigns of 10 subjects each in 2017 and 2018. For each campaign, subjects were randomized into 2 groups: one, the intervention group that received orally daily antioxidant/anti-inflammatory substances during the 60-day bedrest period (Treatment group), and another one (Placebo group) that received a daily water aliquot serving as a control group.

The antioxidant treatment [24] daily dose was composed of 530 mg of polyphenols, 138 mg of vitamin E, 80 µg of selenium, and 2.1 g of omega-3 (Omacor, Laboratoire Pierre Fabre, 92100 Boulogne Billancourt, France) comprising eicosapentaenoic acid (EPA) ethyl ester (EPA: 46%) and docosahexaenoic acid ethyl ester (DHA: 38%).

Before bedrest start, subjects underwent 14 days of baseline data collection (BDC-14 to BDC-1) followed by 60 days of 6° head-down tilt bedrest (HDT1 to HDT60) at the Medes facility. After the end of bedrest, subjects remained in the facility for 14 days of reambulation (R + 1 to R + 14), and returned to the facility several times for follow-up monitoring.

### 2.2. Muscle Biopsy

Prior to bedrest (BDC-6, Pre), shortly before the end of bedrest (HDT56, Post), and 10 days after reambulation (R + 10), skeletal muscle biopsies from the hip vastus lateralis (*VL*) muscle were taken under local anesthesia from the right leg of each subject. Biopsies were obtained according to a well-established experimental protocol using a 5 mm Bergström hollow needle under vacuum suction and in sterile and local anesthesia (1% lidocaine) conditions. For each biopsy, one piece was immediately frozen in liquid nitrogen-cooled isopentane, stored at −80 °C, and dedicated to the immunohistochemical analysis [25]. The other piece was further divided into 2 equal pieces, snap frozen in liquid nitrogen, and stored at −80 °C until use for molecular and biochemical analysis.

### 2.3. Proteomic Analysis

Protein-soluble extracts from frozen *VL* muscle biopsies were obtained from 5 Placebo and 5 Treatment subjects at bedrest Pre, Post, and R + 10. Sample protein extracts were then analyzed by label-free LC–MS/MS and NITRO-DIGE to evaluate proteome and nitrosoproteome changes in pre- and post-BR with and without antioxidant administration and R + 10.

### 2.4. Protein Extraction

Muscle biopsies were ground in a frozen mortar, suspended in HEN buffer (300 mM sucrose, 250 mM 4-(2-hydroxyethyl)-1-piperazineethanesulfonic acid (HEPES); (pH 7.7), 1 mM ethylenediaminetetraacetic acid (EDTA), and 0.1 mM neocuproine), and solubilized by sonication on ice. Sample preparation was carried out in the dark to prevent SNO decomposition. Protein concentration was determined using the bicinchoninic acid (BCA) protein assay kit (Thermo Fisher Scientific, Waltham, MA, USA) and adjusted to 1 mg/mL with HEN buffer.

### 2.5. Label-Free Liquid Chromatography with Tandem Mass Spectrometry (LC–MS/MS)

Sample preparation followed the protocol described in [26]. Protein extracts were reduced for 45 min in 5 mM 1,4-Dithiothreitol (DTT) at 60 °C, carbamidomethylated for 45 min in 15 mM iodoacetamide, and digested with mass spectrometry-grade trypsin gold (Promega, Fitchburg, MA, USA) for 16 h at 37 °C using a protein/trypsin ratio of 50:1. After acidification with trifluoracetic acid and desalting on C18 tips (Zip-Tip C18 micro, Millipore, Burlington, MA, USA), peptide samples were vacuum concentrated, reconstituted in HPLC buffer A (0.1% formic acid), and separated on a Dionex UltiMate 3000 HPLC System with an Easy Spray PepMap RSLC C18 column (250 mm, internal diameter of 75 μm) (Thermo Fisher Scientific); then, the samples adopted a five-step acetonitrile (ACN)/formic acid gradient (5% ACN in 0.1% formic acid for 5 min, 5–35% ACN in 0.1% formic acid for 139 min, 35–60% ACN in 0.1% formic for 40 min, 60–100% ACN for 1 min, 100% ACN for 10 min, at a flow rate of 0.3 μL/min) and were electrosprayed into an Orbitrap Fusion Tribrid (Thermo Fisher Scientific). The LTQ-Orbitrap was operated in positive mode in data-dependent acquisition mode to automatically alternate between a full scan (350–2000 m/z) in the Orbitrap (at resolution 60,000, AGC target 1,000,000) and subsequent collision-induced dissociation (CID) MS/MS in the linear ion trap of the 20 most intense peaks from full scan (normalized collision energy of 35%, 10 ms activation). Isolation window: 3 Da; unassigned charge states: rejected; charge state 1: rejected; charge states 2+, 3+, 4+: not rejected; dynamic exclusion enabled (60 s, exclusion list size: 200). Mass spectra were analyzed using MaxQuant software (version 1.6.3.3; Max Planck Institute of Biochemistry, Martinsried, Germany). The initial maximum allowed mass deviation was set to 6 ppm for monoisotopic precursor ions and 0.5 Da for MS/MS peaks. Enzyme specificity was set to trypsin/P, and a maximum of 2 missed cleavages were allowed. Carbamidomethylation was set as a fixed modification, while *N*-terminal acetylation and methionine oxidation were set as variable modifications. The spectra were searched by the Andromeda search engine against the *Homo sapiens* Uniprot sequence database (74,823 proteins, release 1 January 2020). Protein identification required at least 1 unique or razor peptide per protein group. Quantification in MaxQuant was performed using the built in extracted ion chromatogram (XIC)-based label-free quantification (LFQ) algorithm using fast LFQ. The required false discovery rate (FDR) was set to 1% at the peptide, 1% at the protein, and 1% at the site-modification level, and the minimum required peptide length was set to 7 amino acids.

### 2.6. Identification of S-Nitrosated Proteins by 2-D CyDye-Maleimide DIGE (NITRO-DIGE)

A modified biotin switch method [27] with CyDye maleimide monoreactive sulfhydryl-reactive fluorescent dyes (GE Healthcare, Little Chalfont, UK) to identify SNO proteins was used, as described in [28]. Free thiols were blocked with 4X volume of 50 mM iodoacetamide (IAA) in HEN buffer containing 2.5% SDS for 30 min. Excess IAA was removed by cold acetone precipitation. Protein pellets were washed; dissolved in HEN buffer containing 1% SDS, 5 mM sodium ascorbate, and 1 µM copper sulfate [29]; and incubated at room temperature for 1 h in order to reduce *S*-nitrosothiols. After acetone precipitation, proteins were dissolved in labeling buffer (30 mM Tris-HCl (pH 8), 8 M urea, 4% 3-[(3-cholamidopropyl)dimethylammonio]-1-propanesulfonate (CHAPS) at 2.5 mg/mL. CyDye DIGE Fluor reagent (10 μM Cy3 or Cy5) was added to each sample and incubated at room temperature for 1 h to label NO-released thiols. Each group consisted of at least 3 biological replicates; each replicate was labeled with Cy5, and a mixture containing an equal amount of all samples was labeled with Cy3 as the internal standard. After quenching with 50 mM DTT, labeled samples (internal standard versus each replicate) were mixed and separated by two-dimensional electrophoresis. Samples were run in duplicate using 24 cm, 3–10 non-linear immobilized pH-gradient (IPG) strips. Isoelectric focusing was performed on an IPGphor electrophoresis unit (GE Healthcare) using a gradient ranging from 200 to 8000 V, reaching a total of 75,000 Vh. Focused proteins on IPG strips were prepared for second dimension by reduction and alkylation. Second dimension was carried out using the Ettan Dalt II system (GE Healthcare) on 20 × 25 cm^2^, 12% T, 2.5% C constant concentration polyacrylamide gels at 20 °C and 15 mA.

### 2.7. Image Acquisition and Statistical Analysis

Images from CyDye-labeled gels were acquired by Typhoon 9200 Imager (GE Healthcare), and image analysis was performed by DeCyder software (version 6.5, GE Healthcare, Little Chalfont, UK). For each condition (Placebo and Treatment), 3 groups of subjects were analyzed, and for each group (Pre, Post, and R + 10), only spots present in at least 90% of the samples were considered.

### 2.8. Protein Identification

Protein identification was carried out by matrix-assisted laser desorption/ionization–time-of-flight (MALDI-ToF) mass spectrometry (MS). For protein identification, semi-preparative gels were loaded with unlabeled sample (400 μg per strip); electrophoretic conditions were the same as NITRO-DIGE, and gels were stained with a total-protein fluorescent stain (Krypton, ThermoFisher Scientific). Image acquisition was performed using a Typhoon 9200 laser scanner. Spots of interest were excised from gel using the Ettan spot picker robotic system (GE Healthcare), destained in 50% methanol/50 mM ammonium bicarbonate, and incubated with 30 μL of 6 ng/mL trypsin (Promega) dissolved in 10 mM ammonium bicarbonate for 16 h at 37 °C. Released peptides were subjected to reverse-phase chromatography (Zip-Tip C18 micro, Millipore) and eluted with 50% acetonitrile (ACN)/0.1% trifluoroacetic acid. Peptide mixture (1 μL) was diluted in an equal volume of 10 mg/mL alpha-cyano-4-hydroxycinnamic acid matrix dissolved in 70% ACN/30% citric acid and processed on an Ultraflex III MALDI-ToF/ToF (Bruker Daltonics, Billerica, MA, USA) mass spectrometer. MS was performed at an accelerating voltage of 20 kV, and spectra were externally calibrated using Peptide Mix calibration mixture (Bruker Daltonics); 1000 laser shots were taken per spectrum. Spectra were processed by FlexAnalysis software v. 3.0 (Bruker Daltonics) setting the signal to noise threshold value to 6, and search was carried out by correlation of uninterpreted spectra to *Homo sapiens* entries in Uniprot Proteomes UP5640 20,200,812 (97,065 sequences; 38,762,114 residues) using BioTools v. 3.2 (Bruker Daltonics) interfaced to the online MASCOT software, which utilizes a robust probabilistic scoring algorithm. The significance threshold was set at *p*-value < 0.05. No mass and isoelectric point (pI) constraints were applied, and trypsin was set as enzyme. One missed cleavage per peptide was allowed, and carbamidomethylation was set as fixed modification while methionine oxidation as variable modification. Mass tolerance was set at 30 ppm for MS spectra.

In cases where this approach was unsuccessful, additional searches were performed using electrospray ionization–MS/MS, as previously described [30].

### 2.9. RNA Extraction and cDNA Preparation

Total RNA was isolated from *VL* muscle (*n* = 59 total *VL* muscle biopsies: 20 PRE, 20 HDT58, 19 R + 10) of each subject bed rest group (Placebo, Treatment). Frozen tissue samples were ground to a fine powder under liquid nitrogen, and total RNA was extracted using TRIzol method, following the manufacturer’s instructions and including glycogen co-precipitating step. Reverse transcription was performed on 400 ng of total RNA by using SuperScript VILO complementary DNA (cDNA) Synthesis (ThermoFisher Scientific).

### 2.10. Quantitative Polymerase Chain Reaction (qPCR)

Quantitative PCR was performed by the SYBR Green method, as described elsewhere [31]. The PCR parameters were initial denaturation at 95 °C for 30 s followed by 40 cycles of 10 s at 95 °C and 30 s at the corresponding annealing temperature for the acquisition of fluorescence signal. All samples were processed simultaneously with RNA- and RT-negative controls.

Specific primers for CAT, GCLC, GSR, GPX1, GSRNOR, GSTK1, HMOX1 NQO1, NQO2, SOD2, TXN, and TXNRD1 were designed using Primer3 software (http://frodo.wi.mit.edu/ (accessed on 28 January 2021), Whitehead Institute for Biomedical Research), and their thermodynamic specificity was determined using the blast alignment search tool (BLAST) sequence alignment national center for biotechnology information (NCBI) and vector NTI software (ThermoFisher Scientific). Specific primers for NRF-2, PPIA, B2M, and RPL32 were already published in [5]. Cyclophilin A (PPIA), beta-2-microglobulin (B2M), and ribosomal protein L32 (RPL32) were used as reference genes, and normalization was performed using GeNorm software (V3.5, 2007, https://genorm.cmgg.be/ (accessed on 28 January 2021)). Data are expressed as means ± SEM. Statistical differences between 2 groups were determined by unpaired *t*-test and one-way ANOVA, respectively. In all tests, differences were considered statistically significant at the 0.05 level of confidence.

### 2.11. Statistical Analysis

Label-free LC–MS/MS statistical analyses were performed using the Perseus software (version 1.4.0.6; Max Planck Institute of Biochemistry, Martinsried, Germany). For each experimental group, the proteins identified in at least 75% of samples were considered. Statistically significant differences were computed by ANOVA test and FDR (*p* < 0.05). NITRO-DIGE statistically significant differences were computed by analysis of variance (ANOVA) and corrected for Tukey’s test (*p*-value < 0.05); when the use of ANOVA was not possible, the non-parametric Kruskal–Wallis (*p*-value < 0.05) test was adopted. False discovery rate was applied to correct for multiple tests to reduce the overall error. Statistically changed proteins underwent power analysis, and only spots reaching a sensitivity cut-off > 0.8 were considered as differentially expressed.

For qPCR analysis, data are expressed as means ± SEM. Statistical differences between two groups or multiple comparison were determined by unpaired *t*-test and one-way ANOVA, respectively. In all tests, differences were considered statistically significant at the 0.05 level of confidence.

## 3. Results

### 3.1. Proteomic Analysis

**Figure 1:**
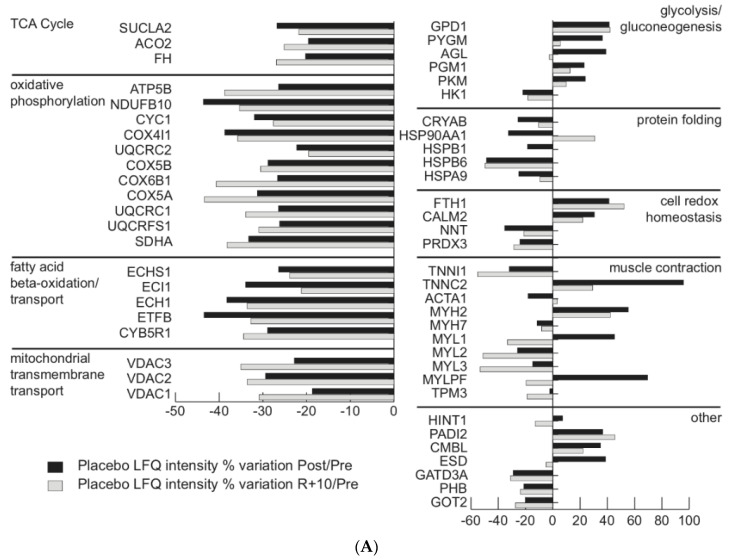

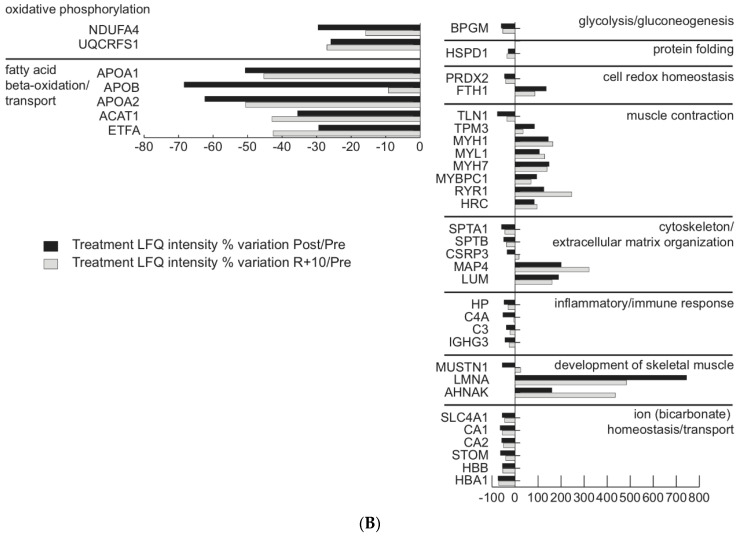
(**A**) Proteomic analysis of vastus lateralis (*VL*) muscles in the Placebo group. Histograms of differentially expressed proteins (label-free quantification (LFQ), intensity % variation, ANOVA and Tukey’s test, *n* = 5, *p* ≤ 0.05) in bedrest Placebo-Post (black bars) and Placebo-R + 10 subjects (gray bars) vs. Placebo-Pre condition. The individual variability within the group (Pre, Post, and R + 10) is shown in Figure S1. (**B**) Proteomic analysis of VL muscles in the Treatment group. Histograms of differentially expressed proteins (LFQ intensity % variation, ANOVA and Tukey’s test, *n* = 5, *p* ≤ 0.05) in bedrest Treatment-Post (black bars) and Treatment-R + 10 subjects (gray bars) vs. Treatment-Pre condition. The individual variability within the group (Pre, Post, and R + 10) is shown in Figure S2.

Label-free (LC–MS/MS) proteomic approach was utilized to identify differentially expressed proteins in muscle tissues from Pre-, Post-, and R + 10-BR subjects (*n* = 5 each time point) without and with antioxidant administration. Label-free LC–MS/MS provided a whole dataset of 904 individual proteins, and 241 were common to Placebo samples and 388 were common to all Treatment samples. Comparing Placebo-Post and Placebo-R + 10 to Placebo-Pre, we found that 54 proteins were changed, whereas comparing Treatment-Post and Treatment-R + 10 to Treatment-Pre, 37 proteins were differentially abundant. In Figure 1A,B, changed proteins were grouped according to their function. Tables S1 and S2 show the list of identified proteins, according to the above-mentioned criteria, by label-free LC–MS/MS analysis.

Figure 1A graphically shows results from label-free LC–MS/MS reported in Table S1 and indicates protein changes in Placebo-Post and Placebo-R + 10 vs. Pre. Decrements were observed in tricarboxylic acid (TCA) cycle, oxidative phosphorylation, fatty acid beta oxidation) and transport proteins both in Post and R + 10 vs. Pre. Glycolysis/gluconeogenesis increased in Post and R + 10 vs. Pre, except hexokinase-1 (HK1), which decreased both in Post and R + 10 vs. Pre and glycogen debranching enzyme (AGL), which changed (i.e., increased in Post and decreased in R + 10 vs. Pre). Proteins involved in protein folding decreased both in Post and R + 10 vs. Pre, with the exception of heat shock protein HSP 90-alpha (HSP90AA1), which decreased in Post vs. Pre and increased in R + 10 vs. Pre. Cell redox homeostasis was influenced with increase of ferritin heavy chain (FTH1) and calmodulin (CALM2) that increased and nicotinamide-adenine-dinucleotide-phosphate (NAD(P)) transhydrogenase (NNT) and peroxiredoxin 3 (PRDX3) that decreased in Post and R + 10 vs. Pre. Muscle contraction was also influenced with some contractile protein that decreased in Post and R + 10 vs. Pre, such as troponin I (TNN1), myosin heavy chain 7 (MYH7), myosin light chain 2 and 3 (MYL2 and MYL3), and tropomyosin 3 (TPM3). At variance, actin (ACTA1) decreased in Post and increased in R + 10 vs. Pre, and myosin light chain 1 (MYL1) and myosin regulatory light chain (MYLPF) increased in Post and decreased in R + 10 vs. Pre. In the group of others, histidine triad nucleotide-binding protein 1 (HINT1) and *S*-formylglutathione hydrolase (ESD) increased in Post and decreased in R + 10 vs. Pre. Protein-arginine deiminase type-2 (PADI2) and carboxymethylene butenolidase (CMBL) increased in both Post and R + 10 vs. Pre, whereas glutamine aminotransferase-like class 1 domain-containing protein 3A (GATD3A), prohibitin (PHB), and aspartate aminotransferase (GOT2) decreased both in Post and R + 10 vs. Pre.

Figure 1B graphically shows results from label-free LC–MS/MS differential analysis reported in Table S2 and indicates levels of proteins that are dysregulated by BR in Treatment-group in both Post and R + 10 vs. Pre. As a result, oxidative phosphorylation, cytochrome c oxidase subunit NDUFA4, and cytochrome b-c1 complex subunit Rieske (UQCRFS1) were decreased. Furthermore, fatty acid oxidation and lipid transport proteins were decreased. The glycolytic protein bisphosphoglycerate mutase (BPGB) and the mitochondrial chaperone protein 60 kDa heat shock protein (HSPD1) were also decreased. Relating to cell redox homeostasis, FTH1 increased and peroxiredoxin-2 (PRDX2) decreased in Post and R + 10 vs. Pre. Altered levels of proteins involved in muscle contraction were found such as slow-twitch TPM3, MYH7, myosin-binding protein C (MYBPC), and fast-twitch myosin-1 (MYH1) and myosin light chain 1/3 (MYL1), which increased in Post and R + 10 vs. Pre. The costameric protein talin-1 (TLN1) also decreased, whereas calcium ion transport proteins ryanodine receptor 1 (RyR1) and sarcoplasmic reticulum histidine-rich calcium-binding protein (HRC) protein increased. Cytoskeletal proteins showed a decrement of spectrin alpha and beta chain (SPTA1, SPTB), whereas a promotor of myogenesis, cysteine, and glycine-rich protein 3 (CSRP3) and microtubule-associated protein (MAP4) protein increased. In addition, the extracellular matrix component lumican (LUM) increased. Transport and inflammatory/immune response proteins were decreased, whereas proteins involved in the development of skeletal muscle such as prelaminin A/C (LMNA) and neuroblast differentiation-associated protein (AHNAK) increased both in Post and R + 10 vs. Pre, with the exception of musculoskeletal embryonic nuclear protein 1 (MUSTN1), which decreased in Post vs. Pre and increased in R + 10 vs. Pre.

**Figure 2:**
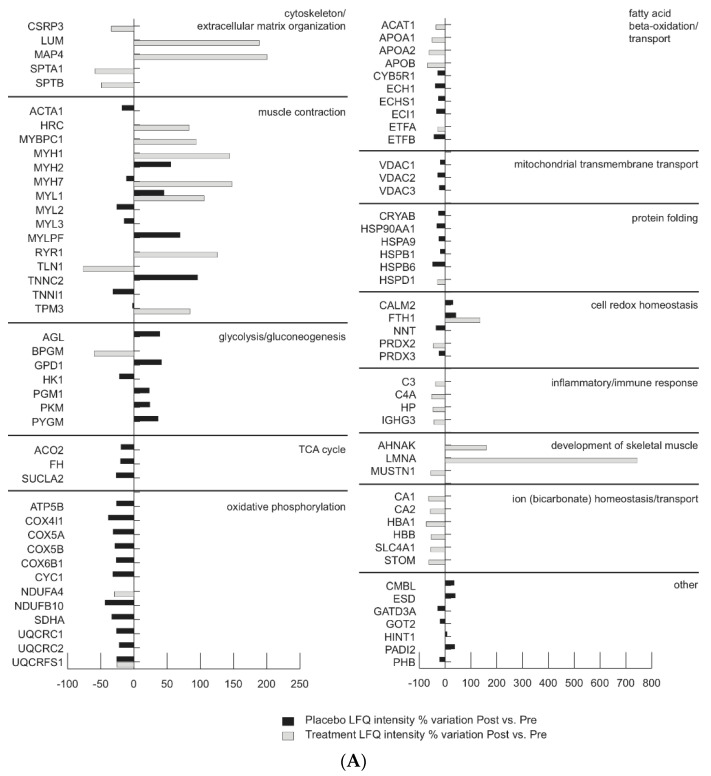

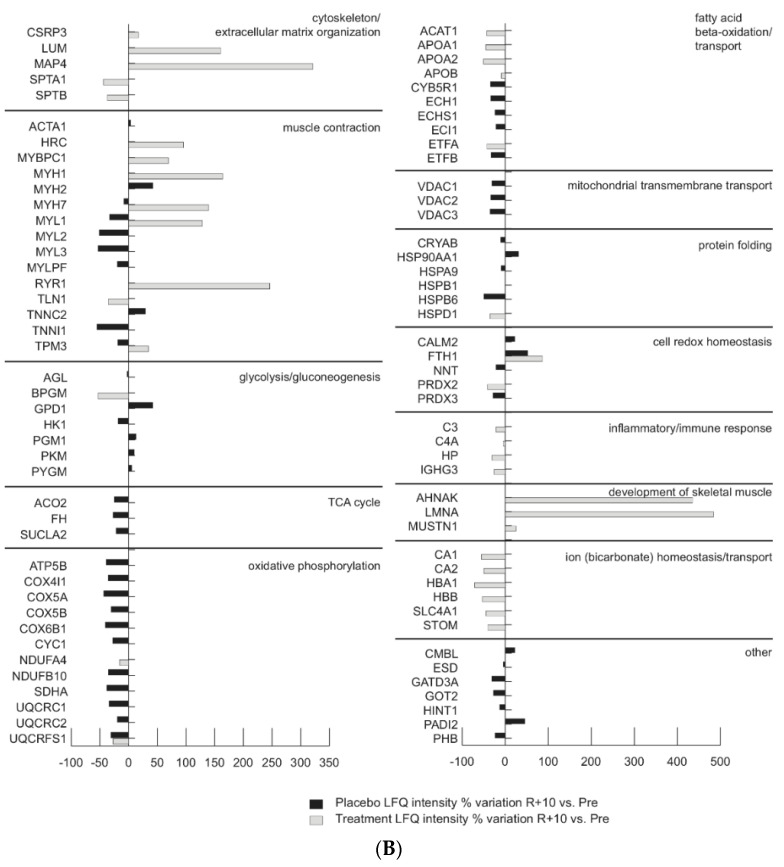
(**A**) Comparison of Placebo (black bars) and Treatment (gray bars) proteomic datasets in bedrest Post vs. Pre condition (LFQ intensity % variation, ANOVA and Tukey’s test, *n* = 5, *p* < 0.05). (**B**) Comparison of Placebo (black bars) and Treatment (gray bars) proteomic datasets in bedrest R+10 vs. Pre condition (LFQ intensity % variation, ANOVA and Tukey’s test, *n* = 5, *p* < 0.05).

Figure 2A,B graphically shows comparative results from label-free LC–MS/MS differential analysis reported in Tables S1 and S2 and highlight levels of proteins dysregulated or unchanged by BR in Treatment vs. Placebo groups in Post vs. Pre (2A) and in Treatment-versus Placebo groups in R + 10 vs. Pre (2B). Results indicate that in the treated group, proteins from cytoskeletal and extracellular matrix (CSRP3, LUM, MAP4) were increased, whereas SPTA1 and SPTB decreased compared to untreated subjects. Proteins involved in muscle contraction (HRC, MYBPC1, MYH1, MYL1, RYR1, TPM3) increased as well as proteins involved in the development of skeletal muscle (AHNAK, LMNA) and MUSTN1 decreased. Those changes were absent in the Placebo group. Results indicate that glycolysis (except BPGM, which decreased), TCA cycle, oxidative phosphorylation, fatty acid beta-oxidation, and mitochondrial transmembrane transport were unchanged in treated subjects whereas they were decreased in the Placebo group. Proteins involved in protein folding were also unchanged compared to the Placebo, whereas proteins involved in ion homeostasis were decreased. These results highlight at molecular levels the effects of the treatment in BR subjects that are retained at R + 10.

The individual variability within the group (Pre, Post, and R + 10) of differentially expressed proteins in the analyzed subjects under Placebo and Treatment is shown in Figures S1 and S2, respectively.

**Figure 3:**
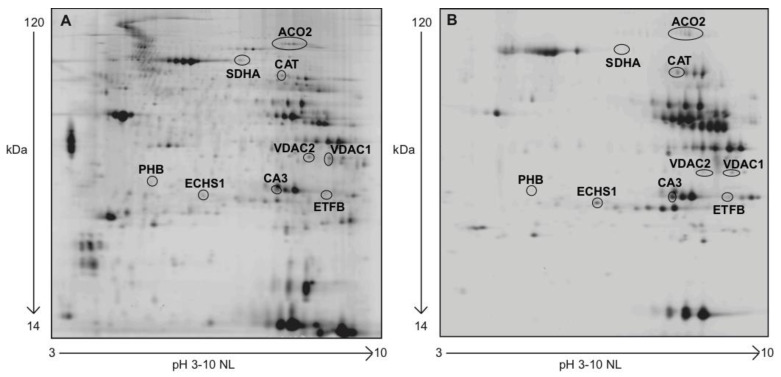
Representative *VL* muscle protein nitroprofiles. (**A**) Individual 2D pattern gel image of 40 μg of protein extract, separated in a pH 3–10 non-linear immobilized pH-gradient (IPG) in the first dimension, and SDS gel (12% T, 2.5% C) in the second dimension. (**B**) NITRO-DIGE gel image of 20 μg of protein extract, separated in a pH 3–10 non-linear IPG strip in the first dimension and SDS gel (12% T, 2.5% C) in the second dimension.

An overview of representative profiles of 2D-DIGE (panel A) and NITRO-DIGE (panel B) for the identification of *S*-nitrosated protein spots is shown in Figure 3. Among 101 nitrosated proteins, 9 of them were differentially nitrosated in Post and R + 10 vs. Pre in Placebo and Treatment. The differentially nitrosated proteins were aconitase 2 (ACO2), succinyl-CoA dehydrogenase (SDHA), catalase (CAT), voltage dependent anion channel 1 and 2 (VDAC1, VDAC2), enoyl-CoA hydratase (ECHS1), carbonic anhydrase 3 (CA3), electron transfer protein (ETFB), and prohibitin (PHB).

**Figure 4:**
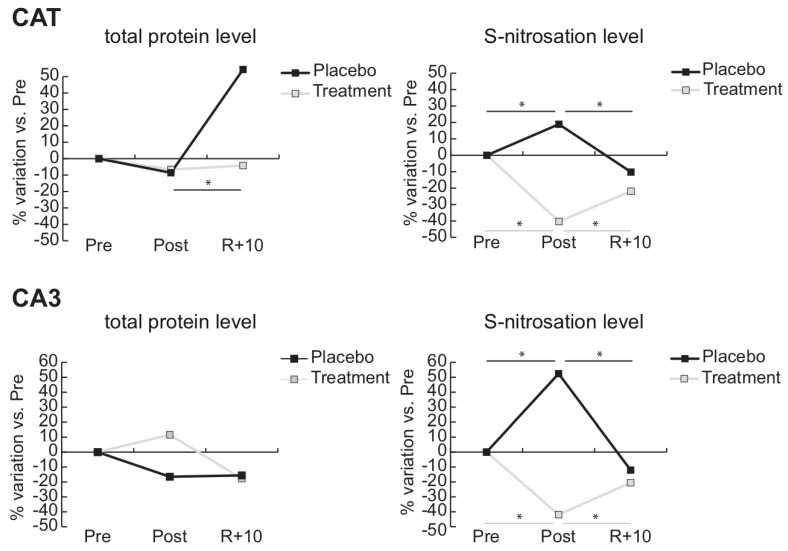
Total abundance and *S*-nitrosated protein level of catalase (CAT) and carbonic anhydrase 3 (CA3). Line charts illustrating protein abundance and *S*-nitrosation level variations (%) of cytosolic proteins catalase (CAT) and carbonic anhydrase 3 (CA3) in post-bedrest (Post) and recovery (R + 10) compared to pre-bedrest (Pre) condition in untreated controls (Placebo, black lines) or in subjects under antioxidant treatment (Treatment, gray lines) (* = significant difference, ANOVA and Tukey, *n* = 5, *p*-value ≤ 0.05).

Line charts of the protein differential abundance and protein nitrosation of cytosolic proteins as catalase (CAT) and carbonic anhydrase (CA3) are shown in Figure 4. Specifically in Placebo, protein levels of CAT were unchanged in Post and increased in R + 10, whereas the nitrosation levels of CAT increased in Post and decreased in R + 10; thus, an increment of nitrosation occurred under BR and was indicative of an altered redox homeostasis. In treated subjects, the total protein level remained unchanged, whereas the nitrosation levels decreased. Concerning CA3, in Placebo, the protein levels decreased, although this was not statistically significant in Post and remained lower in R + 10, while nitrosation increased in Post and returned to a lower level in R + 10. During Treatment, levels of nitrosation remained lower, despite the tendential increment of protein levels.

**Figure 5:**
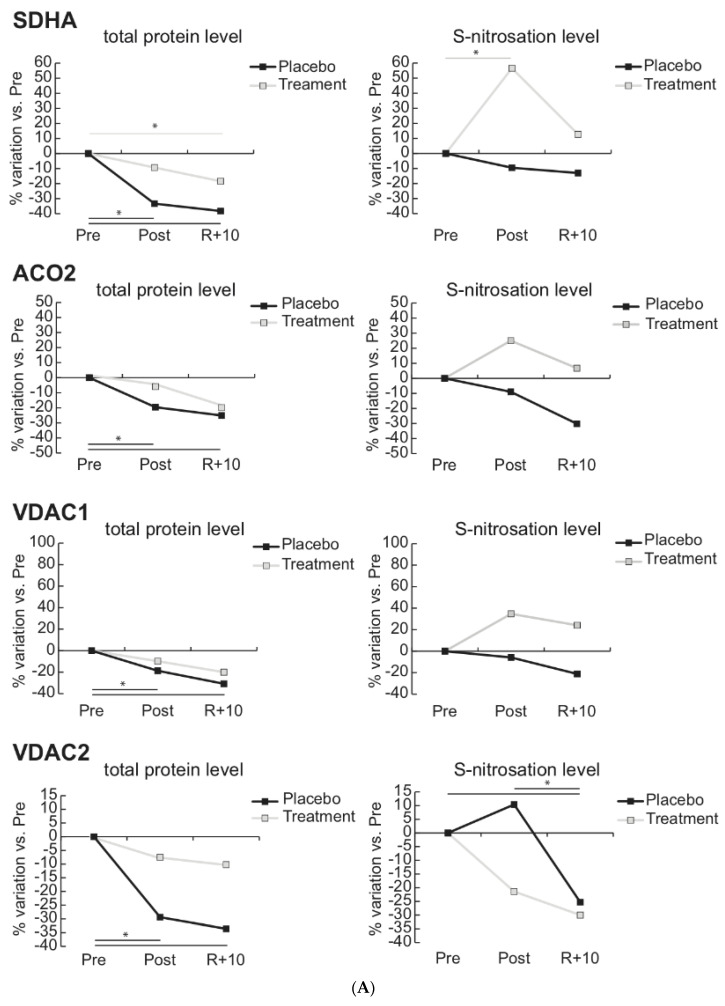

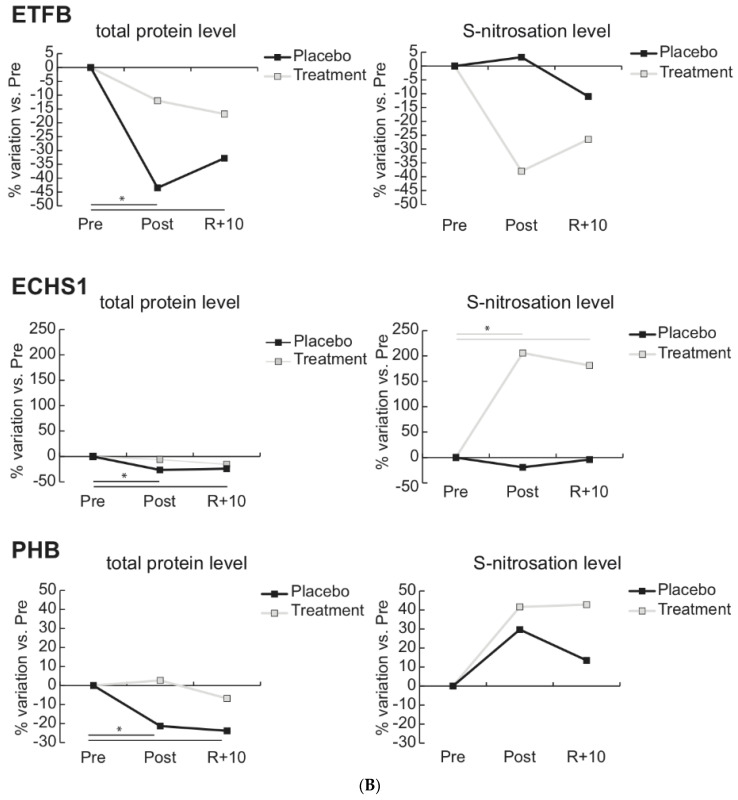
(**A**,**B**) Total abundance and *S*-nitrosated protein level of mitochondrial proteins. Line charts illustrating protein abundance and *S*-nitrosation level variations (%) of succinate dehydrogenase (ubiquinone) flavoprotein subunit (SDHA), aconitate hydratase (ACO2), voltage-dependent anion-selective channel protein 1 and 2 (VDAC1, VDAC2), electron transfer flavoprotein subunit beta (ETFB), enoyl-CoA hydratase (ECHS1), and prohibitin (PHB) in post-bedrest (Post) and recovery (R + 10) compared to pre-bedrest (Pre) condition in untreated controls (Placebo, black lines) or in subjects under antioxidant treatment (Treatment, gray lines) (* = significant difference, ANOVA and Tukey, *n* = 5, *p*-value ≤ 0.05).

Line charts of the levels of differentially abundant proteins involved in mitochondrial homeostasis and of their nitrosation level are shown in Figure 5A,B. In Placebo, SDHA, ACO2, VDAC1 and 2, ETFB, ECHS1, and PHB decreased in Post and remained decreased after recovery, suggesting impairment of the mitochondrial machinery. The nitrosation levels followed the tendency of decrement in Post and R + 10 for SDHA, ACO2, VDAC1, and ECHS1, while VDAC2, ETFB, and PHB showed increased levels of nitrosation (although not statistically significant).

Interestingly, under Treatment, the total level of these proteins increased compared to Placebo, and nitrosation levels tended to decrease (VDAC2, ETFB) or followed the same trend (PHB), suggesting a role of the Treatment in the maintenance of mitochondrial homeostasis in agreement with proteomic data.

Concerning the inter-individual variability, results of tyrosine nitration of muscle extracts in all subjects enrolled in this campaign are shown in supplementary Figure S3. Levels of tyrosine nitration performed by immunoblotting were changed with a high inter-individual variability, both in Placebo and Treatment.

### 3.2. Antioxidant Responsive Element (ARE) Enzyme qPCR Analysis in Pre- and Post-BR and R + 10 VL Muscle Biopsies

Nuclear translocation of Nrf-2 leads to increased transcription levels of a large set of antioxidant enzymes involved in the antioxidant defense response such as the antioxidant responsive elements (ARE) or phase II protein detoxification enzymes [32]. Thus, a subset of ARE enzymes were investigated in the present study by qPCR in *VL* muscle biopsies (Table 1).

**Figure 6:**
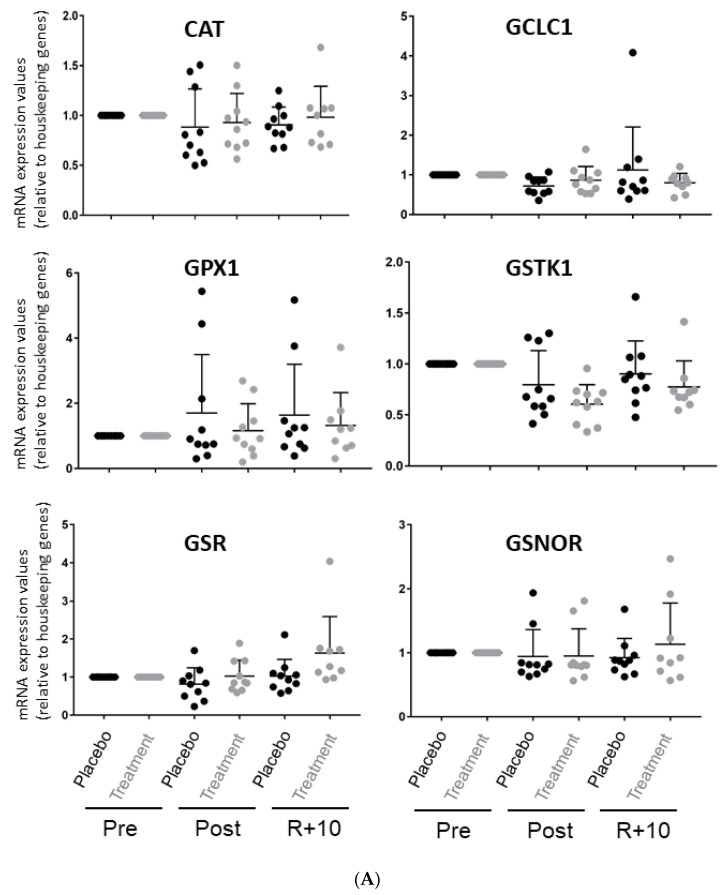

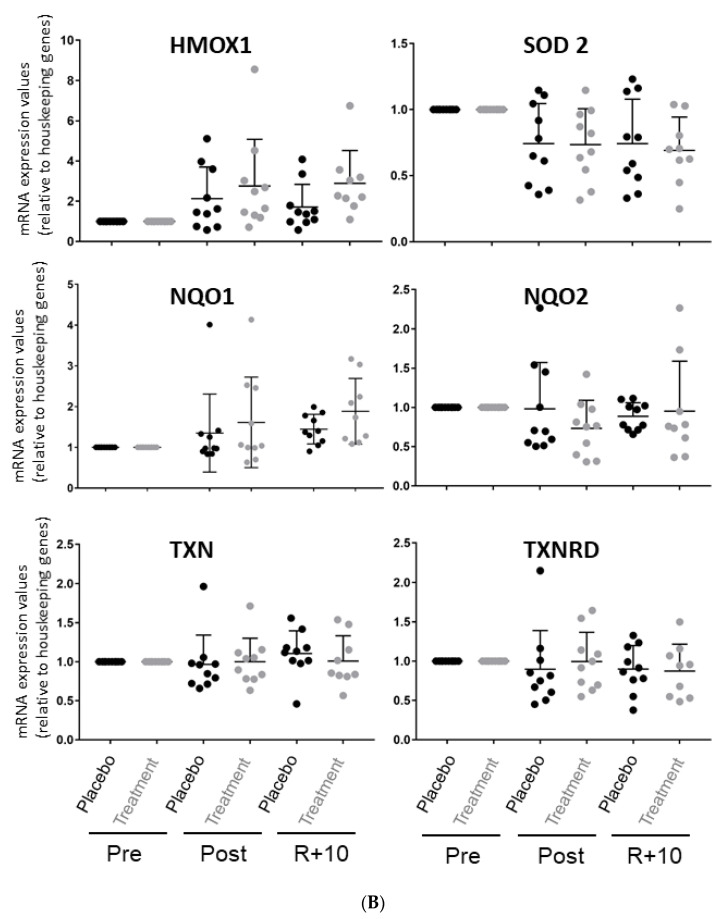
(**A**,**B**) Antioxidant responsive element (ARE) gene quantitative PCR (qPCR) analysis in *VL* muscle biopsies. (**A**) Scatter plots showing the relative mRNA expression levels of CAT, GCLC, GPX1, GSTK1, GSR, and GSNOR; (**B**) scatter plots showing the relative mRNA expression levels of HOMX1, SOD2, NQO1, NQO2, TXN, and TXNRD in *VL* muscle biopsies before (Pre), after (Post) bedrest (BR) and 10 days after ambulation or recovery (R + 10). Investigated ARE gene mRNA levels of each subject enrolled in the two BR campaigns (*n* = 10 for each campaign) are shown (Significant difference, ANOVA and *t*-test, *n* = 10, *p*-value ≤ 0.05). The graph represents the mean ± SEM.

Figure 6A,B show the qPCR results of 12 ARE genes analyzed in *VL* muscle of each group. Table 1 lists the gene codes of the specific primers utilized and changes in post-BR and R + 10 vs. pre-BR (up or down expression as indicated by one or two arrows) in Placebo vs. Treatment.

Although only trends have been observed, the results suggest that four of the shortlisted ARE enzymes (CAT, GCLC1, NQO2, SOD2) showed a trend toward decrease in the Treatment group. Notably, two of the redox pathway key enzymes, nicotinamide dinucleotide phosphate NAD(P)H-dehydrogenase 1 (NQO1) and heme oxygenase (HOMX1), showed a trend toward an increase after BR (Table 1). The shortlist of the antioxidant defense enzymes investigated is reported in Table 1.

Taken together, these results suggest that during extended periods of physical inactivity (long-term bedrest) there is an increase in oxidative/nitrosative stress, which affects specific antioxidant defense enzymes such as NQO1, GPX1, and HOMX1 at the transcriptional level. Notably, administration of the antioxidants as nutrition treatment in bedrest enhances this response, at least for NQO1 and HOMX1.

### 3.3. Nuclear Factor Erythroid 2-Related Factor 2 (Nrf-2) Quantitative PCR (qPCR) Analysis in Pre- and Post-BR and Rec VL Muscle Biopsies

The Nrf2 transcription factor plays a key role in the antioxidant response element (ARE)-mediated gene expression. Thus, the Nrf2 mRNA expression levels were investigated by qPCR experiments in Post and R + 10 vs. Pre subjects for both Placebo and Treatment groups.

**Figure 7:**
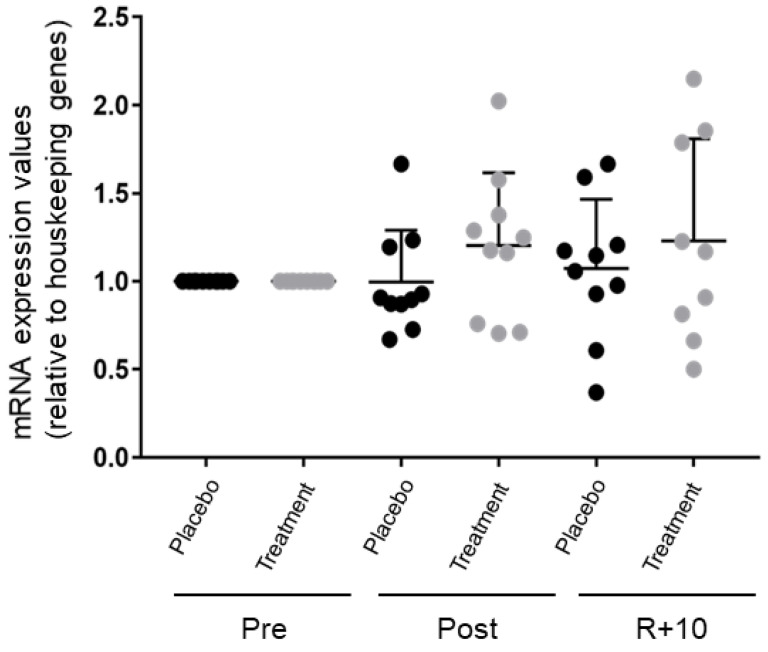
Nuclear factor erythroid 2-related factor 2 (Nrf-2) quantitative PCR analysis in *VL* muscle biopsies. Scatter plots showing the relative mRNA expression levels of Nrf-2 in *VL* muscle biopsies before (Pre) and after (Post) BR and 10 days after ambulation or recovery (R + 10). Nfr-2 mRNA levels of each subject enrolled in the two BR campaigns (*n* = 10 for each campaign) are shown (Significant difference, ANOVA and *t*-test, *n* = 10, *p*-value ≤ 0.05). The graph represents the mean ± SEM.

Figure 7 shows no significant Nrf-2 mRNA level changes in the Placebo group after BR, whereas an increased trend was observed in the Treatment group. These results well correlate with the slight enhancement of Nrf2 accumulation in myonuclei of treated subjects (Figure S4). However, due to higher inter-individual variability, these changes were not statistically significant. Thus, concerning Nrf-2 transcript, only a trend towards increase was observed after nutritional antioxidant treatment in bedrest.

## 4. Discussion

The main findings from the 2017–2018 Toulouse Cocktail Study reported in the present study suggest that a targeted nutritional intervention (albeit in absence of exercise) is promising, not only to promote muscle recovery at the protein level but also to induce post-transcriptional changes of functional muscle proteins in healthy male subjects in long-term bedrest.

At the protein level, treatment of bedrest subjects with the antioxidant cocktail prevented redox homeostasis dysregulation and promoted structural remodeling (TPM3, MYH7, MYBPC, MYH1, MYL1, HRC, and LUM). Our findings were further supported by increase of the intracellular calcium release channel protein (RyR1), a positive regulator of myogenesis (CSRP3), and changes in a set of other proteins involved in skeletal muscle development (MUSTN1, LMNA, AHNAK) highlight the efficacy of the antioxidant cocktail as nutrition countermeasure intervention in our experimental model. In more detail, our results indicate that chronic BR disuse decreased cellular physiological processes related to metabolic pathways, protein folding, and regulatory proteins of the muscle contraction machinery. Glycolysis/gluconeogenesis and cell redox homeostasis were increased, and in contrast, mitochondrial protein synthesis and redox homeostasis were decreased after BR, suggesting a reduced redox regulation of the mitochondrial function that was recovered by the treatment. These results correlate well with NITRO-DIGE data. In Placebo subjects, *S*-nitrosation levels of CA3 and CAT proteins were increased, whereas mitochondrial protein levels decreased as their nitration levels (SDHA, ACO2, VDAC1, ECHS1), with the exception of VDAC2, ETFB, and PHB that, despite a decreased abundance, showed a trend towards increased nitrosation. Nitrosation levels of these proteins were unchanged or tendentially decreased in treated subjects, despite total protein levels being higher compared to the Placebo group.

VDAC2, ETFB, and PHB play fundamental roles in mitochondrial homeostasis and, in some way, resume the impact of nitrosative stress and its recovery in the mitochondrial machinery. VDACs are pore-forming proteins predominantly found in the outer mitochondrial membrane, and VDAC2 may facilitate mitochondrial Ca^2+^ uptake. In the heart, it is involved in the coupling with RyR2 to transfer Ca^2+^ from the SR to mitochondria [33]. In our study, we could speculate that the increment of RyR1 observed in cocktail-treated subjects and the decrement of VDAC2 nitration can be associated with the improvement of Ca^2+^ uptake. Further studies, however, are required to investigate this mechanism in more detail. Another result of the study is the increase of nitrosated PHB in Placebo Post and R + 10. PHB2/PHB1 are active in a broad range of cellular processes, including cellular signaling, senescence, apoptosis, and mitochondrial biogenesis [34,35,36]. Recently they have been associated to mitophagy. The mitophagy function of the PHB complex (PHB1/2) may have protective effects against aging and neuromuscular diseases. It has been also postulated that it can act concurrently as a regulator of mitochondrial function, promoting the binding of the mitochondrial inner membrane to the autophagy cargo receptor LC3 [37]. Thus, the downregulation of the nitroso PHB levels observed in cocktail-treated subjects could be associated with the recovery, not only of the mitochondrial function but also of mitophagy. Studies to determine the level of mitophagy in our subjects are currently ongoing.

Another important result from this molecular study is the HSP90 decrement observed in Placebo Post vs. Pre and the recovery of protein level in R + 10. The decrease of the ATP-dependent molecular chaperone Hsp90 has been associated with muscle cell dysfunction [38]. Recently, it has been reported that this molecule is associated with a rapid myosin heavy chain replacement. The inhibition of HSP90 in myotubes decreases the replacement rate of myosin, indicating that the activity of HSP90 is critical for promoting fiber regeneration [39]. Levels of this protein were restored in R + 10 and dysregulation was not observed in subjects under treatment.

Concerning ETFB, the subunit beta is located in the mitochondrial matrix and mediates transfer of electrons from mitochondrial flavoenzymes to the respiratory chain for ATP production and is involved in fatty acid metabolism and amino acid catabolism. Carbonylation and oxidative modifications have been identified by proteomic analysis, indicating the susceptibility of this protein to oxidative damage that contribute to accumulation of intermediate metabolites, as well as to a decrease in ATP production [40]. Furthermore, nitration of CAT was significantly decreased, suggesting that the treatment counteracted the nitrosative stress.

Notably, antioxidant treatment enhanced Nrf-2 myonuclear localization and the transcription of antioxidant response element (Nrf-2 ARE) enzymes/signaling pathways involved in the first step of detoxification such as heme oxygenase 1 (HOMX1) and quinone oxydoreductase 1 (NQO1). However, due to the inter-individual variability, changes on many of the 12 investigated ARE and on Nrf-2 transcript levels were not statistically significant. Nonetheless, a clear trend towards elevated levels in Treatment vs. Placebo group was present.

In response to chemically induced oxidative stress, cells elicit a large repertoire of cytoprotective antioxidant enzymes (ARE) in their promoter region (nucleotide sequence) that are specifically induced by the activation of Nrf-2. Noteworthy, disruption of Nrf-2/ARE signaling in a mouse model [41] induced endoplasmic reticulum stress [42], leading to unfolded protein response activation [43] and impairment of antioxidant mechanisms responsible for differential cell degradation highlighting the central role of nitrosation redox homeostasis.

The unbalanced regulation of redox state that likely happens during extended period of disuse promotes several skeletal muscle pathophysiologic conditions including atrophy [44].

The coordinated regulation of Nrf-2 signaling pathway is central and essential for preservation of the redox homeostasis in skeletal muscle physiology. Nrf-2 data previously reported by Brocca et al. [45] showed that there was an increase in Nrf-2 mRNA transcriptional activity after 24 days of bedrest, which was not seen after 60 days of bedrest [5], suggesting a time-dependent activation of the Nrf-2 antioxidant defense enzyme system during chronic disuse.

Previously, antioxidant treatments were proposed to improve muscle performance during training exercise, increasing resistance to fatigue, as reviewed by Hernandéz et al. [46]. However, many discrepancies are present in the literature concerning the use of antioxidants as nutrition treatment to prevent oxidative stress-induced skeletal muscle injury/atrophy [9].

Results from the present study, particularly those provided at the post-transcriptional level, can be considered a hint for the comprehension of mechanisms involved in the response to BR and antioxidant treatments. Our results indicate that BR induces a profound dysregulation in the ordered architecture, regulating muscle function that can be prevented by treatments described in the present and previous studies [47,48,49]. These results confirm recent protocols in which a combination of therapies involving exercise countermeasures and nutritional supplementation are suggested, targeting metabolism, mitochondrial homeostasis, and nitrosative stress with the aim of contributing to muscle proteostasis balance to counteract muscle sarcopenia.

However, further studies addressing the role of translational modifications in larger cohorts adopting less invasive procedures utilizing bodily fluids [28] will be required to define these changes more precisely in the context of BR.

We are aware of the limitations of this study, which involves a restricted number of subjects from whom small muscle biopsies were available. Nonetheless, these results provide a better understanding of dysregulation occurring in muscle tissue associated with a loss of muscle mass in BR and on the effects of oxidative/nitrosative stress.

Understanding the molecular mechanisms regulating the antioxidant defense expression pattern related to either extended periods of muscle disuse or due to chronic exposure to simulated or real microgravity (µG) will enhance our ability to optimize and design potential therapeutic strategies. This may further contribute to establish a “non-invasive countermeasure protocol” in addition to “physical exercise” as a countermeasure intervention, resulting in synergistic effects for maintenance of neuro-musculoskeletal performance, and muscle structure and function during long-term exposure to real µG, for example, in future deep space exploration missions (Moon, Mars). The achievement of such aims may provide the basis for transferring the knowledge from the bed rest analogue on the ground to spaceflight, and finally provides potential benefit to patients with chronic musculoskeletal diseases in different clinical settings.

## 5. Conclusions

In the present study, some positive effects of treating healthy male subjects with the antioxidant cocktail in long-term BR as nutritional countermeasure were found in a large set of functional key muscle proteins including their post-transcriptional levels. In particular, we identified changes in the muscle proteome and in the nitrosative redox homeostasis during chronic BR that were prevented in Treatment vs. Placebo (water) groups. In addition, decreased antioxidant nitrosative defense response was mitigated by treatment with the antioxidant cocktail, suggesting positive effects of the nutritional intervention protocol in BR. In absence of physical exercise, however, nutritional intervention by antioxidant cocktail alone may not be sufficient to prevent structural atrophy in chronically disused skeletal muscles. Further study should include in their protocols anti-oxidative treatment and physical exercise.

## Figures and Tables

**Table 1:** Primer sequences for qPCR analysis and resulting changes in gene transcription.

				Post		Recovery	
Gene Name/Accession Number		Primer	Sequence (5′ → 3′)	Placebo	Treatment	Placebo	Treatment
Chloramphenicol acetyltransferase NM_001752.3	CAT	forward reverse	GATAGCCTTCGACCCAAGCA TTGGAGCACCACCCTGATTG				
Glutamate-cysteine ligase, catalytic subunit NM_001498.3	GCLC1	forward reverse	TTGGTCCTGTCTGGGGAGAA TCCACTGGGTTGGGTTTGAC	↓	↓		↓
Glutathione peroxidase 1 NM_000581.3	GPX1	forward reverse	TATCGAGAATGTGGCGTCCC TCTTGGCGTTCTCCTGATGC	↑		↑	↑
*S*-Nitrosoglutathione reductase NM_000671.4	GSNOR	forward reverse	TAAAGTGGCTGGTGCTTCCC CTACTCCAACCACGACGCTG				↑
Glutathione reductase NM_000637.4	GSR	forward reverse	CACACATCCTGATCGCCACA GGAGAACTTCAGCACCTCCA				↑↑
Glutathione *S*-transferase kappa 1 NM_015917.2	GSTK1	forward reverse	TTGGCTCCACCATAAGGCA GGGGGTAGGGGAAAGACAGA	↓	↓↓	↓	↓
Heme oxygenase 1 NM_002133.2	HOMX1	forward reverse	AGACTGCGTTCCTGCTCAAC GGGGGCAGAATCTTGCACT	↑	↑↑	↑	↑↑
NAD(P)H dehydrogenase [quinone] 1 NM_000903.2	NQO1	forward reverse	CAAAAGAAGCTGGAAGCCGC CATGGCAGCGTAAGTGTAAGC	↑	↑↑	↑	↑↑
NAD(P)H dehydrogenase [quinone] 2 NM_001290221.1	NQO2	forward reverse	CGGGCTGCTTAGGTTGGCA CCAAGGACCGCTCTAGGAGT		↓		
Nuclear factor erythroid 2-related factor 2 NM_006164.4	NRF2	forward reverse	CACAGAAGACCCCAACCAGT CTGTGCTTTCAGGGTGGTTT				
Superoxide dismutase 2 NM_000636.3	SOD2	forward reverse	GGGGTTGGCTTGGTTTCAAT TGCAGTACTCTATACCACTACAAAA	↓	↓	↓	↓↓
Thioredoxin NM_003329.3	TXN	forward reverse	TTCCATCGGTCCTTACAGCC TCCTGACAGTCATCCACATCTAC				
Thioredoxin reductase 1 NM_182729.2	TXNRD1	forward reverse	TCTTAGGACGGTCGGGC TATTGGGCTGCCTCCTTAGC				↓

Arrow: indicates direction of the trend of change. Two arrows: more pronounced compared to one arrow change.

## Data Availability

Study data from this human study other than those published in this work are under privacy regulations but can be obtained on a case-to case basis upon reasonable request from the corresponding author. See also “MDPI Research Data Policies” at https://www.mdpi.com/ethics (accessed on 28 January 2021).

## Supplementary Materials

The following are available online at https://www.mdpi.com/2076-3921/10/3/378/s1: Figure S1. Box-plots of all differentially expressed proteins in Placebo bedrest subjects representing individual variability within the group (Pre, Post, and R + 10). LFQ = label-free quantification. Figure S2. Box-plots of all differentially expressed proteins in Treatment bedrest subjects representing individual variability within the group (Pre, Post, and R + 10). LFQ = label-free quantification. Figure S3. Tyrosine-nitrated protein detection. Enhanced chemiluminescence (ECL) detection in monodimensional acrylamide gel 12% T 4% C, followed by immunobloting with monoclonal anti-3-nitrotyrosine clone 18G4 (Sigma-Aldrich). Results normalized with the total protein-binding dye for membrane SYPRO Ruby. Two main bands are detected with the antibody. (A,B) Graphics of Placebo and Treatment subjects upper band. (C,D) Graphics of Placebo and Treatment subjects lower band. Normalization on pre-bed rest values. Figure S4. Anti-oxidative master gene Nrf-2 regulation in *VL* of bedrest subjects. (a) Representative image Nrf-2 immunopositive myonuclei (green) in *VL* before (Pre) and after (Post) bedrest in Placebo and Treatment groups. Dystrophin (red) used as reference marker to label the subsarcolemmal plasma membrane border; nuclei were counterstained with 4′,6-diamidino-2-phenylindole (DAPI, blue). Inset, magnification Nrf-2-positive myonuclei for comparison with Nrf-2-negative myonuclei and nuclei outside the myofiber. (b) Quantitative analysis of Nrf-2-positive myonuclei in *VL*. The total number of Nrf-2-positive myonuclei was decreased in both *VL* Placebo (Pre, 18.95 ± 3.56%; Post, 2.38 ± 0.94%; *p* ≤ 0.001) and Treatment (Pre, 23.4 ± 4.43%; Post, 8.27 ± 1.94%; *p* ≤ 0.05) group. However, the comparison between both post-BR groups was threefold less decreased in the Treatment Post group (* = significant difference, ANOVA and *t*-test, *n* = 10, *p*-value ≤ 0.05). The graph represents the mean ± SE. Table S1. List of identified proteins in Placebo group by label-free LC–MS/MS analysis. Table S2. List of identified proteins in Treatment group by label-free LC–MS/MS analysis.
