# Supplementary material for: Nitrosative Redox Homeostasis and Antioxidant Response Defense in Disused Vastus lateralis Muscle in Long-Term Bedrest (Toulouse Cocktail Study)
Source: Antioxidants (Basel). 2021 Mar 3;10(3):378. doi: 10.3390/antiox10030378 (PMC8001160; doi:10.3390/antiox10030378)
Supplement: Supplementary file 1 [file antioxidants-10-00378-s001.zip › antioxidants-1112224-supp-revise/Figure S1.pdf]

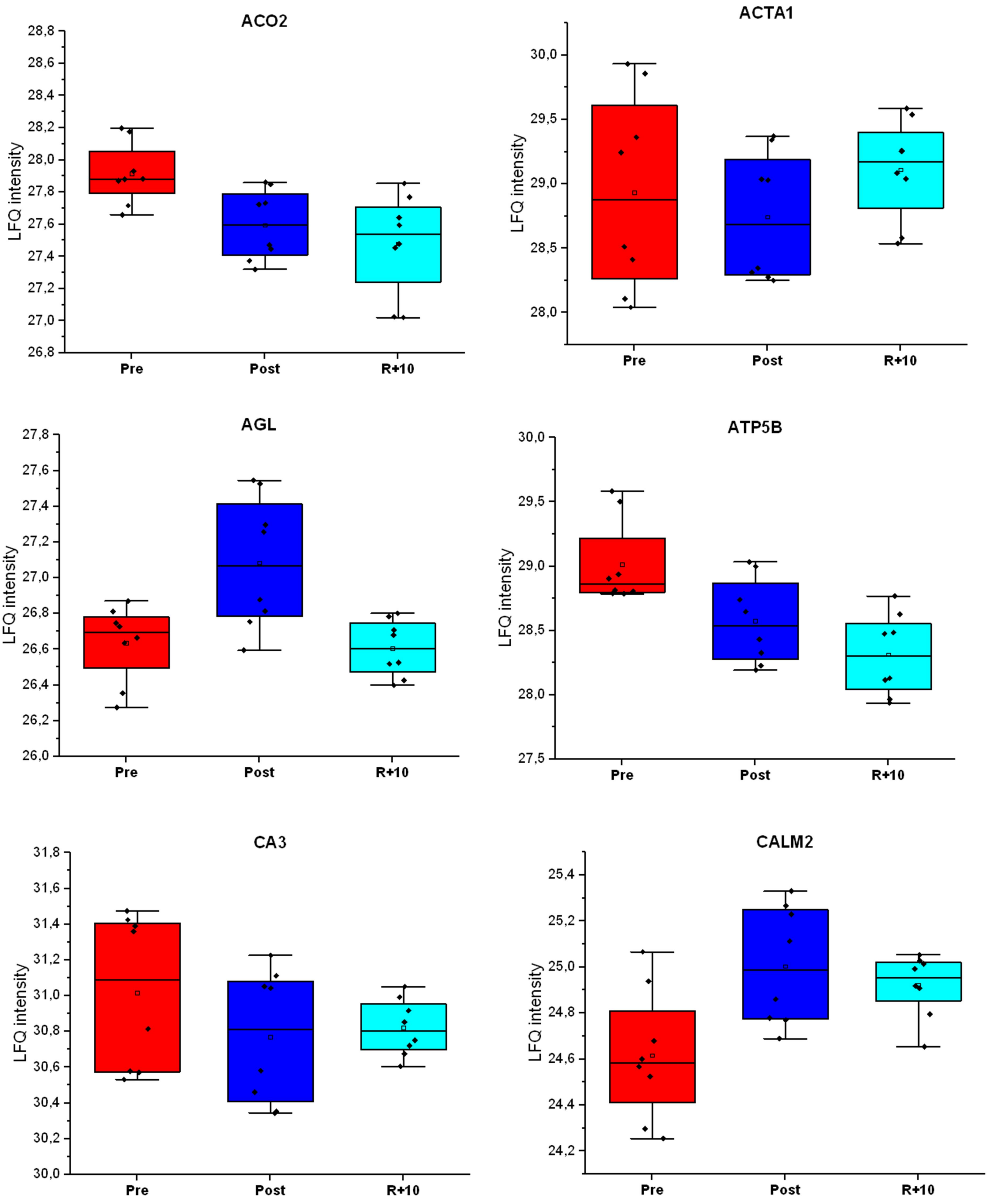
ACO2
LFQ intensity
28,8
28,6
28,4
28,2
28,0
27,8
27,6
27,4
27,2
27,0
26,8
Pre
Post
R+10
ACTA1
LFQ intensity
30,0
29,5
29,0
28,5
28,0
Pre
Post
R+10
AGL
LFQ intensity
27,8
27,6
27,4
27,2
27,0
26,8
26,6
26,4
26,2
26,0
Pre
Post
R+10
ATP5B
LFQ intensity
30,0
29,5
29,0
28,5
28,0
27,5
Pre
Post
R+10
CA3
LFQ intensity
31,8
31,6
31,4
31,2
31,0
30,8
30,6
30,4
30,2
30,0
Pre
Post
R+10
CALM2
LFQ intensity
25,4
25,2
25,0
24,8
24,6
24,4
24,2
Pre
Post
R+10

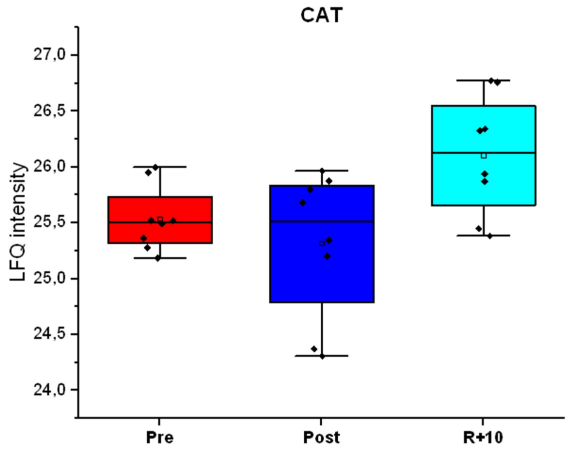
CAT
LFQ intensity
27,0
26,5
26,0
25,5
25,0
24,5
24,0
Pre
Post
R+10

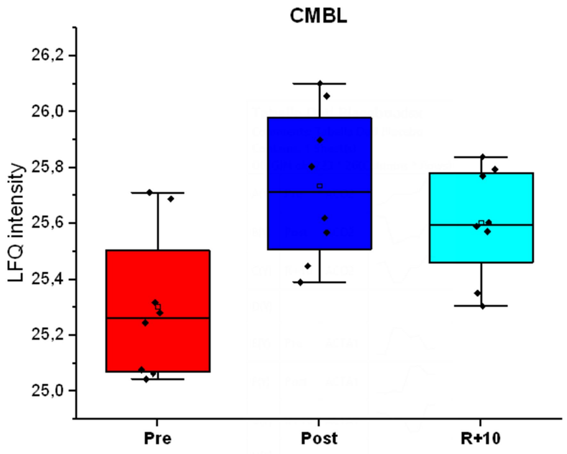
CMBL
LFQ intensity
26,2
26,0
25,8
25,6
25,4
25,2
25,0
Pre
Post
R+10

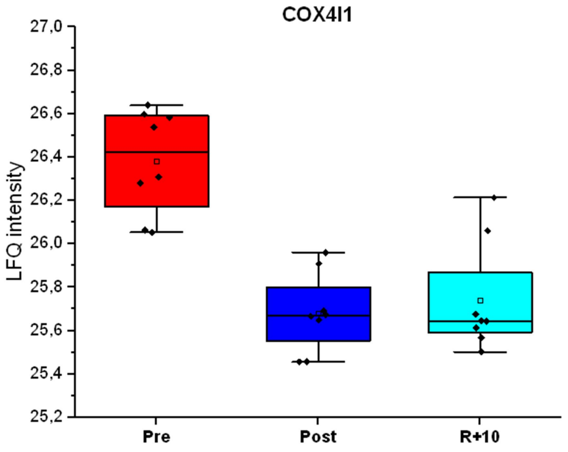
COX4I1
LFQ intensity
27,0
26,8
26,6
26,4
26,2
26,0
25,8
25,6
25,4
25,2
Pre
Post
R+10

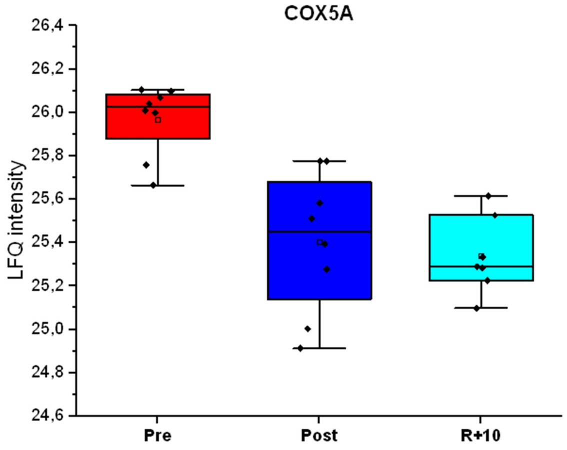
COX5A
LFQ intensity
26,4
26,2
26,0
25,8
25,6
25,4
25,2
25,0
24,8
24,6
Pre
Post
R+10

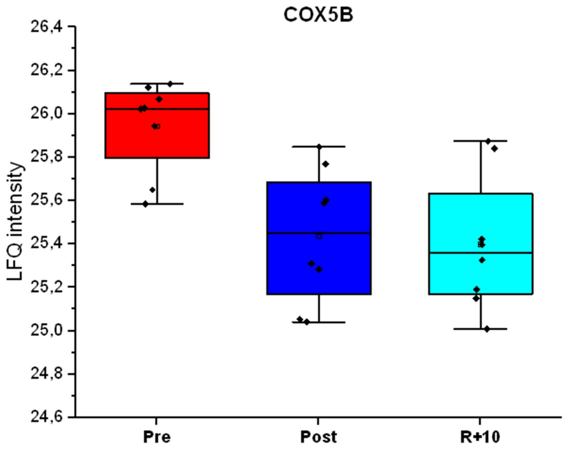
COX5B
LFQ intensity
26,4
26,2
26,0
25,8
25,6
25,4
25,2
25,0
24,8
24,6
Pre
Post
R+10

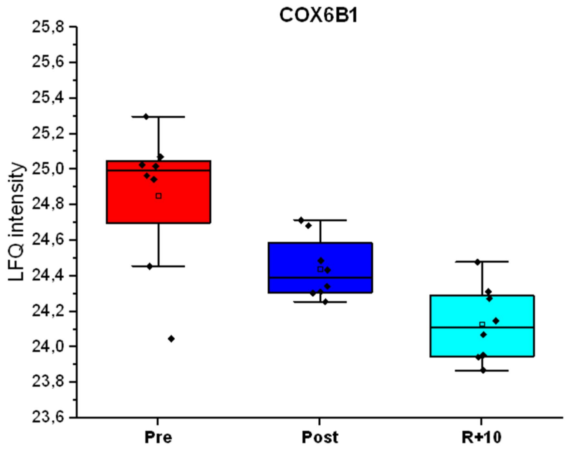
COX6B1
LFQ intensity
25,8
25,6
25,4
25,2
25,0
24,8
24,6
24,4
24,2
24,0
23,8
23,6
Pre
Post
R+10

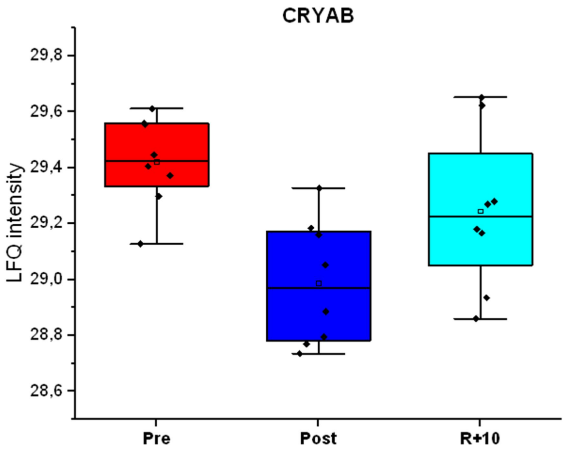
CRYAB
LFQ intensity
29,8
29,6
29,4
29,2
29,0
28,8
28,6
Pre
Post
R+10

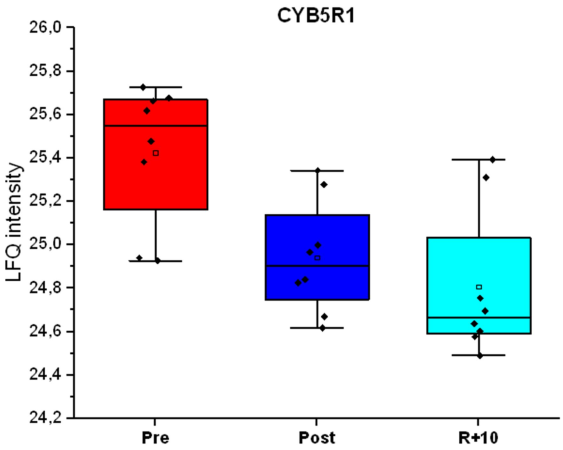
CYB5R1
LFQ intensity
26,0
25,8
25,6
25,4
25,2
25,0
24,8
24,6
24,4
24,2
Pre
Post
R+10

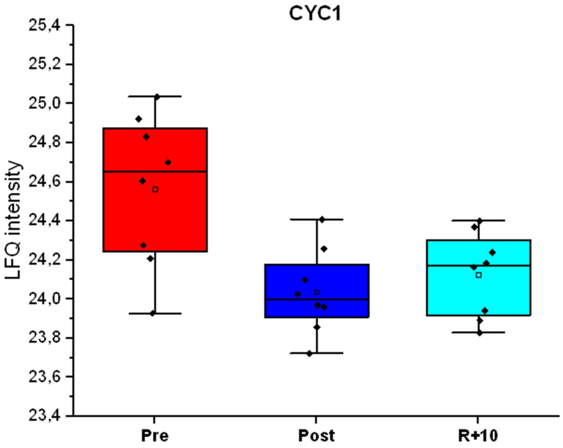
CYC1
LFQ intensity
25,4
25,2
25,0
24,8
24,6
24,4
24,2
24,0
23,8
23,6
23,4
Pre
Post
R+10

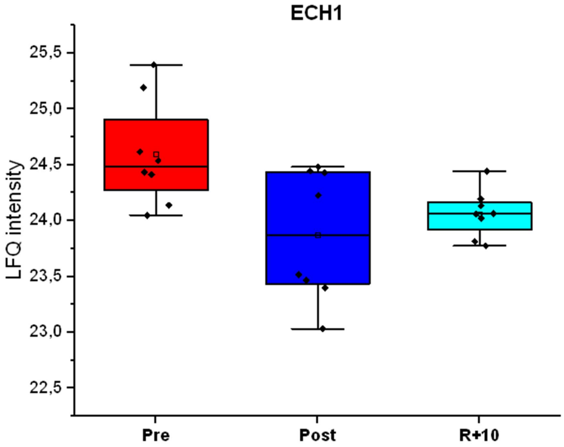
ECH1
LFQ intensity
25,5
25,0
24,5
24,0
23,5
23,0
22,5
Pre
Post
R+10

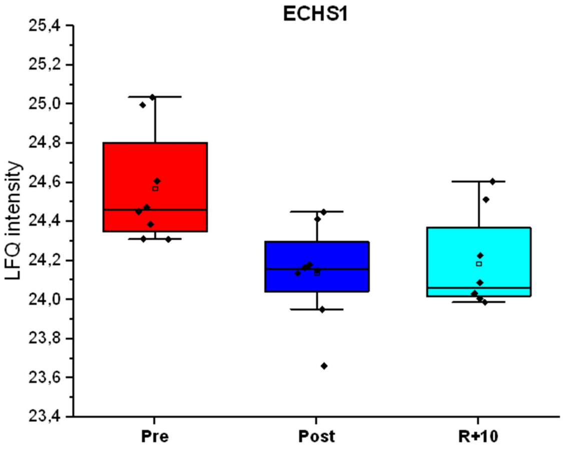
ECHS1
LFQ intensity
25,4
25,2
25,0
24,8
24,6
24,4
24,2
24,0
23,8
23,6
23,4
Pre
Post
R+10

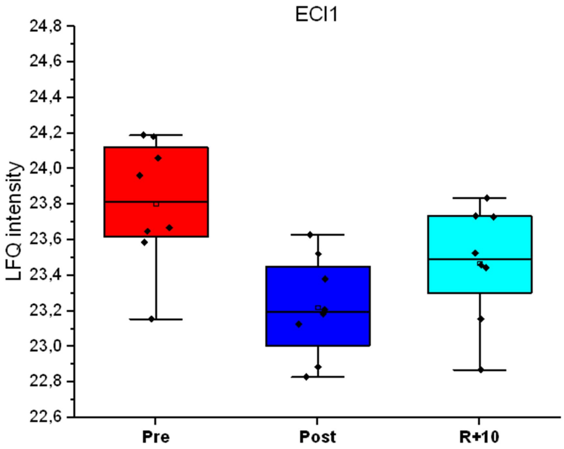
ECI1
LFQ intensity
24,8
24,6
24,4
24,2
24,0
23,8
23,6
23,4
23,2
23,0
22,8
22,6
Pre
Post
R+10

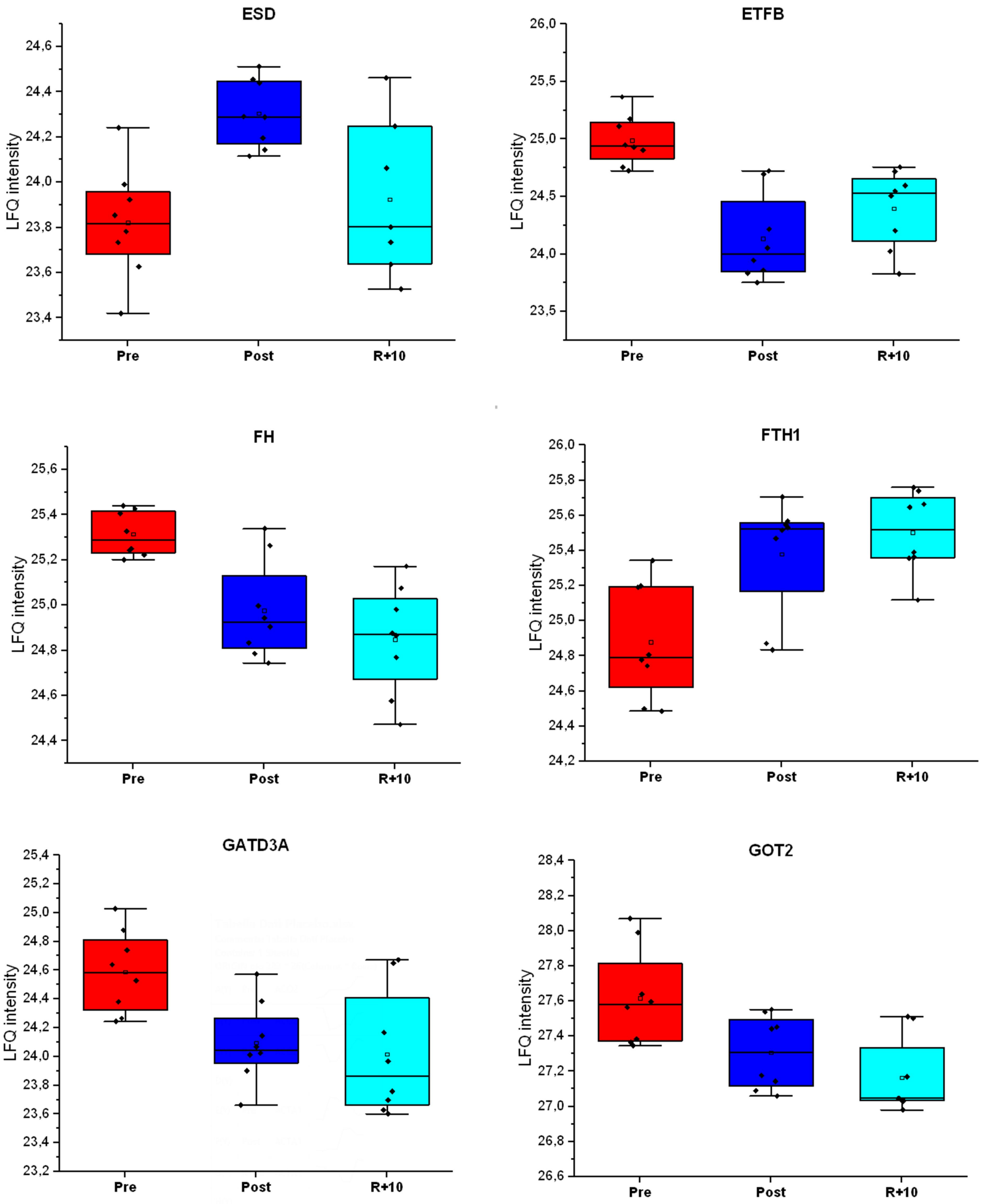
ESD
ETFB
FH
FTH1
GATD3A
GOT2
LFQ intensity
Pre
Post
R+10

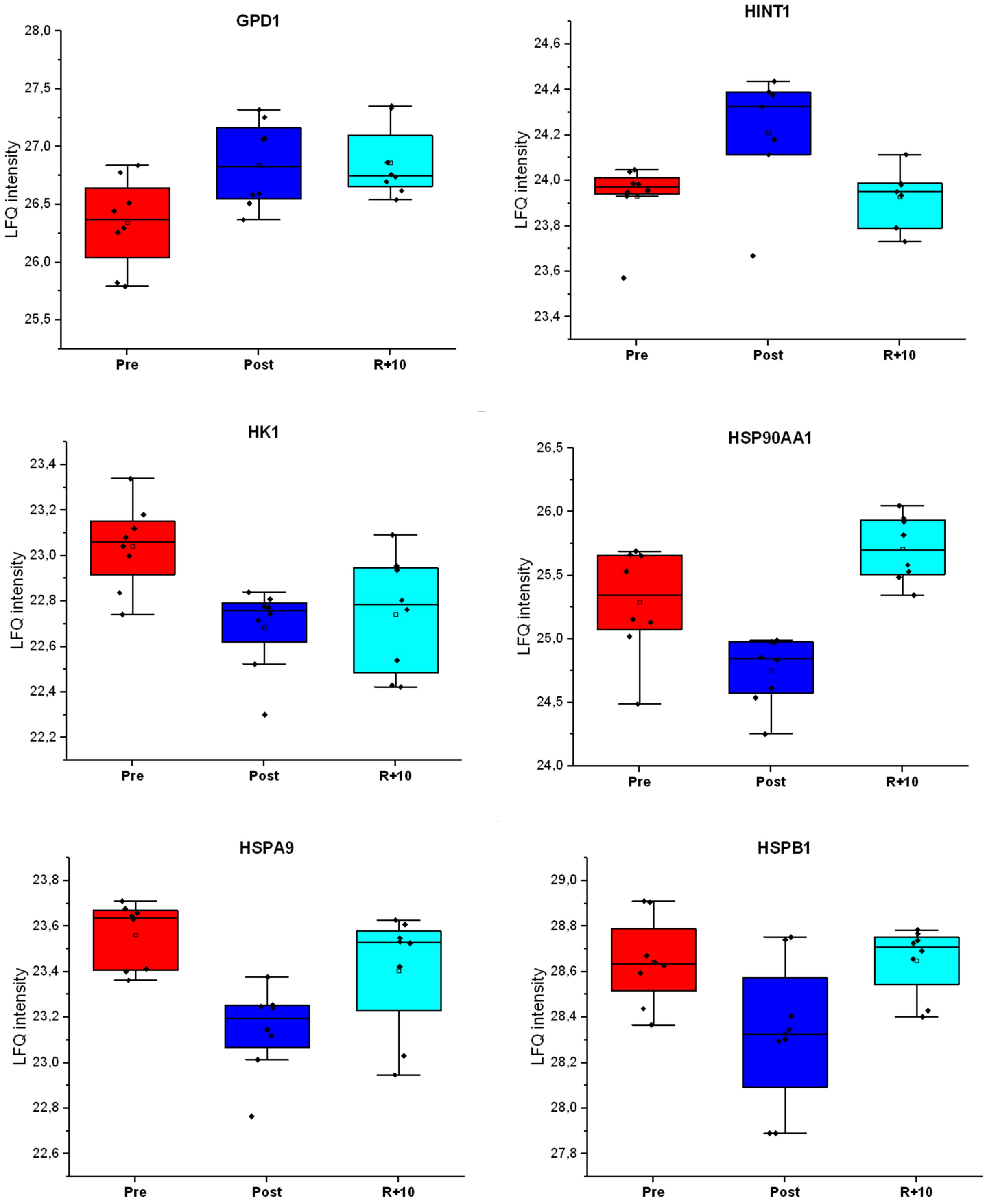
GPD1
LFQ intensity
28,0
27,5
27,0
26,5
26,0
25,5
Pre
Post
R+10
HINT1
LFQ intensity
24,6
24,4
24,2
24,0
23,8
23,6
23,4
Pre
Post
R+10
HK1
LFQ intensity
23,4
23,2
23,0
22,8
22,6
22,4
22,2
Pre
Post
R+10
HSP90AA1
LFQ intensity
26,5
26,0
25,5
25,0
24,5
24,0
Pre
Post
R+10
HSPA9
LFQ intensity
23,8
23,6
23,4
23,2
23,0
22,8
22,6
Pre
Post
R+10
HSPB1
LFQ intensity
29,0
28,8
28,6
28,4
28,2
28,0
27,8
Pre
Post
R+10

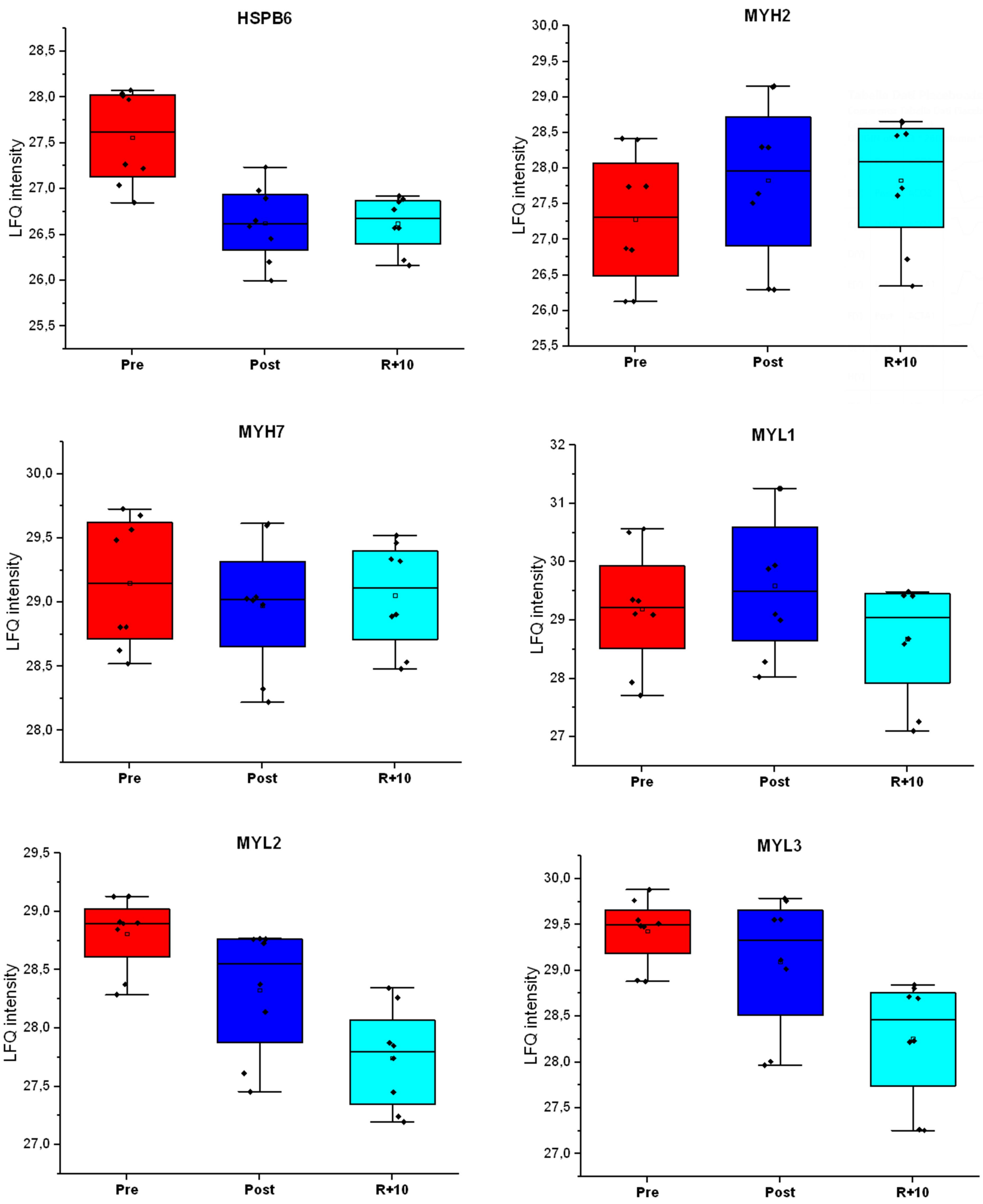
HSPB6
LFQ intensity
28,5
28,0
27,5
27,0
26,5
26,0
25,5
Pre
Post
R+10
MYH2
LFQ intensity
30,0
29,5
29,0
28,5
28,0
27,5
27,0
26,5
26,0
25,5
Pre
Post
R+10
MYH7
LFQ intensity
30,0
29,5
29,0
28,5
28,0
Pre
Post
R+10
MYL1
LFQ intensity
32
31
30
29
28
27
Pre
Post
R+10
MYL2
LFQ intensity
29,5
29,0
28,5
28,0
27,5
27,0
Pre
Post
R+10
MYL3
LFQ intensity
30,0
29,5
29,0
28,5
28,0
27,5
27,0
Pre
Post
R+10

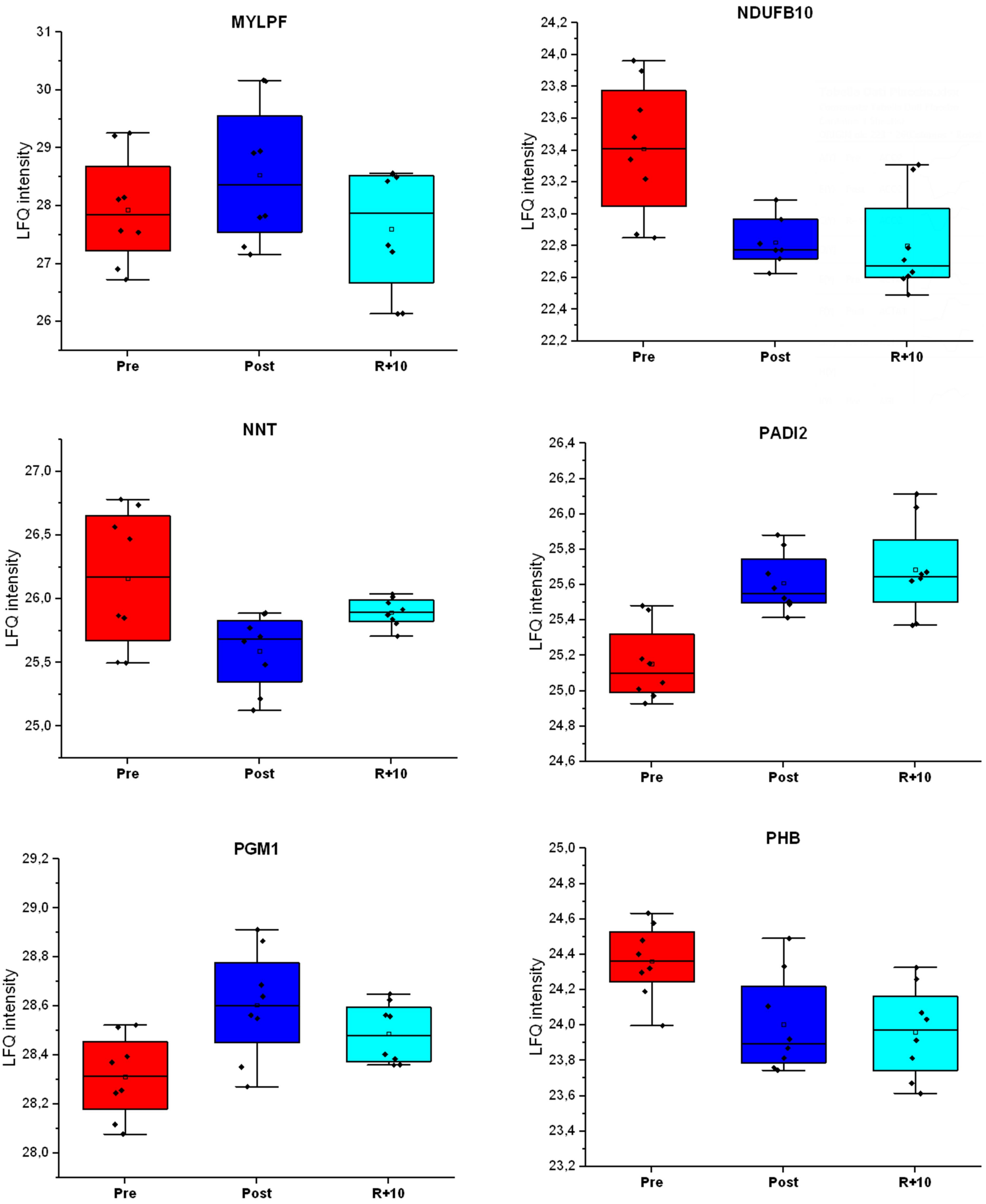
MYLPF
LFQ intensity
31
30
29
28
27
26
Pre
Post
R+10
NDUFB10
LFQ intensity
24,2
24,0
23,8
23,6
23,4
23,2
23,0
22,8
22,6
22,4
22,2
Pre
Post
R+10
NNT
LFQ intensity
27,0
26,5
26,0
25,5
25,0
Pre
Post
R+10
PADI2
LFQ intensity
26,4
26,2
26,0
25,8
25,6
25,4
25,2
25,0
24,8
24,6
Pre
Post
R+10
PGM1
LFQ intensity
29,2
29,0
28,8
28,6
28,4
28,2
28,0
Pre
Post
R+10
PHB
LFQ intensity
25,0
24,8
24,6
24,4
24,2
24,0
23,8
23,6
23,4
23,2
Pre
Post
R+10

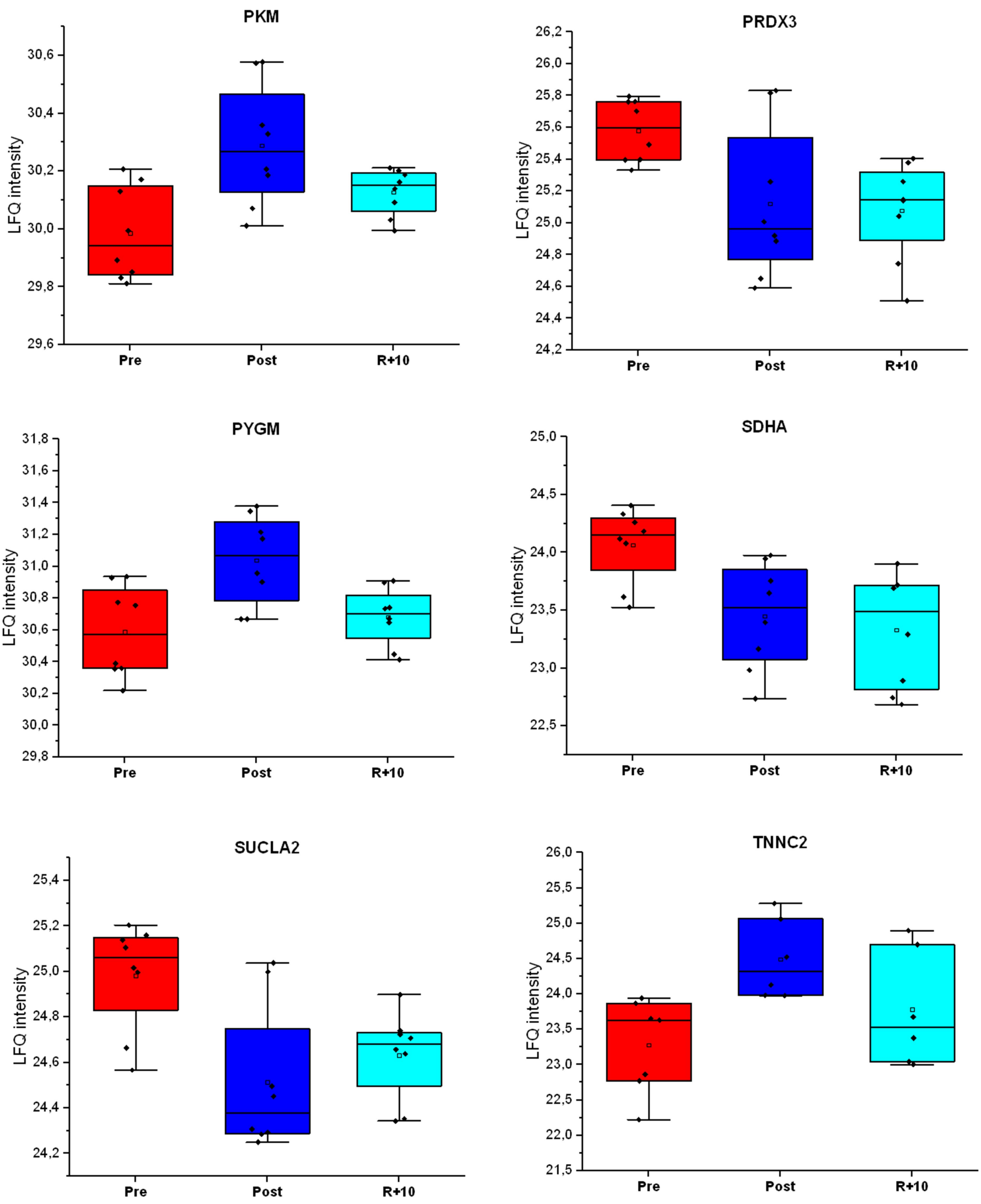
PKM
30,6
30,4
30,2
30,0
29,8
29,6
LFQ intensity
Pre
Post
R+10
PRDX3
26,2
26,0
25,8
25,6
25,4
25,2
25,0
24,8
24,6
24,4
24,2
LFQ intensity
Pre
Post
R+10
PYGM
31,8
31,6
31,4
31,2
31,0
30,8
30,6
30,4
30,2
30,0
29,8
LFQ intensity
Pre
Post
R+10
SDHA
25,0
24,5
24,0
23,5
23,0
22,5
LFQ intensity
Pre
Post
R+10
SUCLA2
25,4
25,2
25,0
24,8
24,6
24,4
24,2
LFQ intensity
Pre
Post
R+10
TNNC2
26,0
25,5
25,0
24,5
24,0
23,5
23,0
22,5
22,0
21,5
LFQ intensity
Pre
Post
R+10

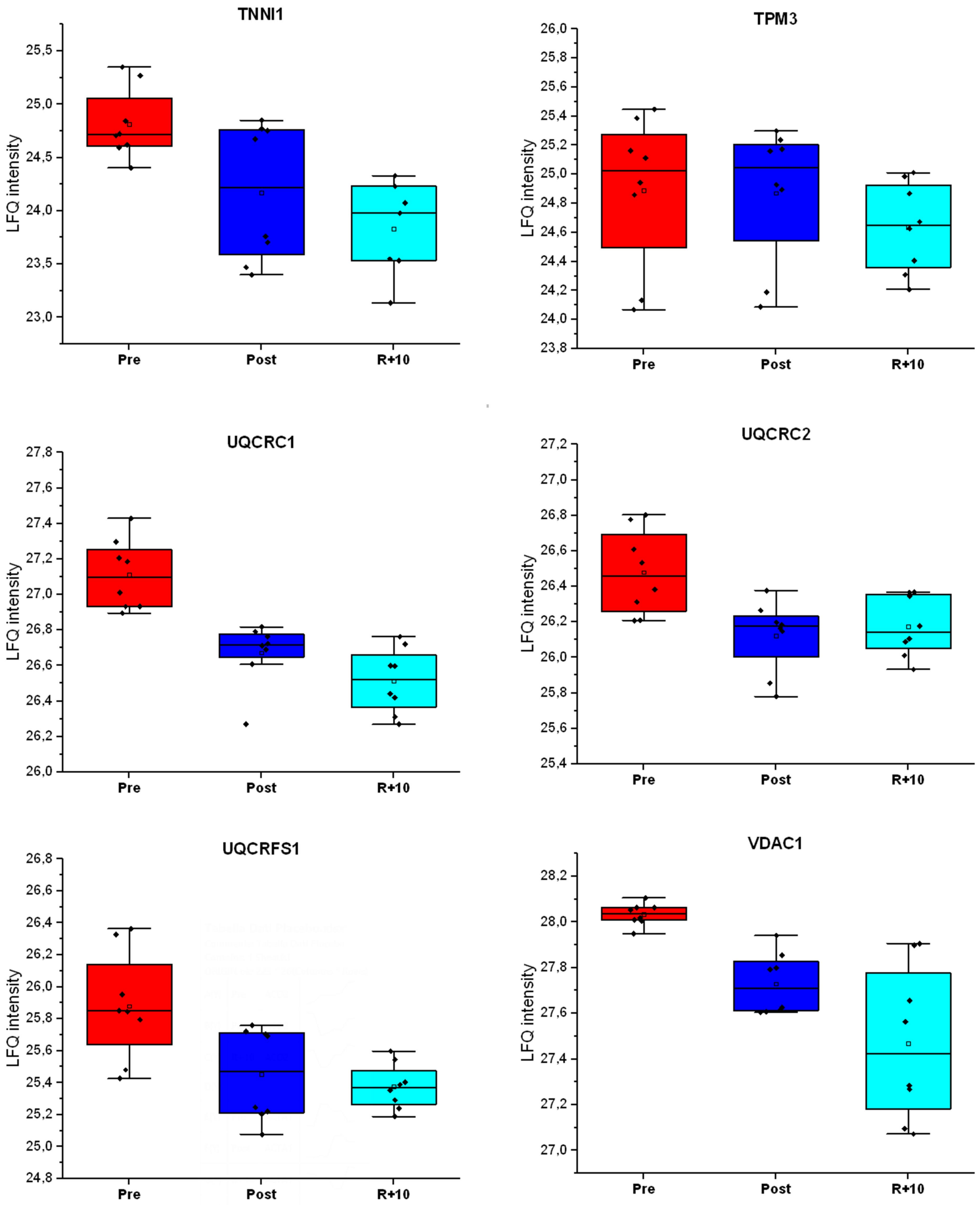
TNNI1
TPM3
UQCRC1
UQCRC2
UQCRFS1
VDAC1
LFQ intensity
Pre
Post
R+10

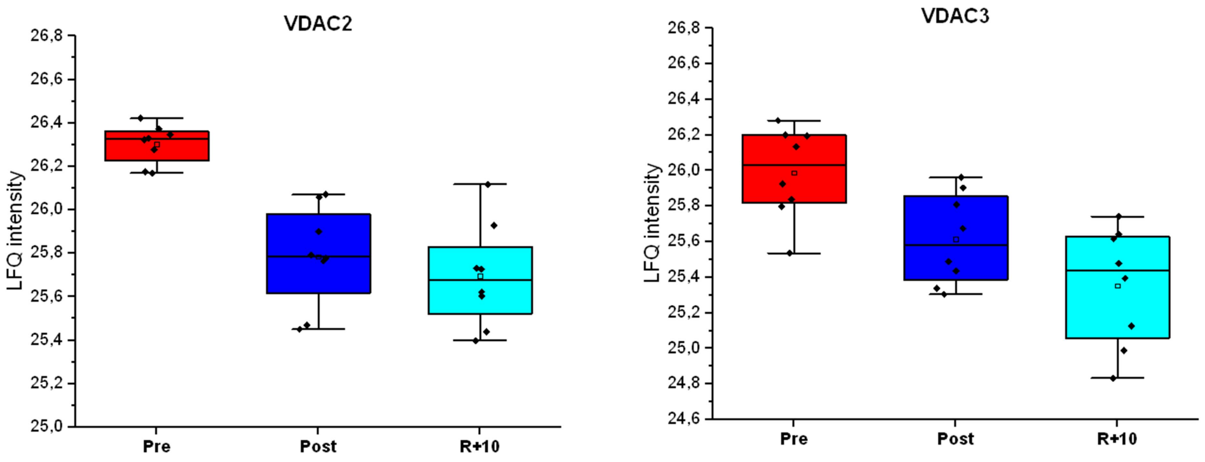

Figure S1. Box-plots of all differentially expressed proteins in Placebo bed rest subjects representing individual variability within the group (Pre, Post and R+10). LFQ = Label-free quantification.
