# Supplementary material for: Nitrosative Redox Homeostasis and Antioxidant Response Defense in Disused Vastus lateralis Muscle in Long-Term Bedrest (Toulouse Cocktail Study)
Source: Antioxidants (Basel). 2021 Mar 3;10(3):378. doi: 10.3390/antiox10030378 (PMC8001160; doi:10.3390/antiox10030378)
Supplement: Supplementary file 1 [file antioxidants-10-00378-s001.zip › antioxidants-1112224-supp-revise/Figure S2.pdf]

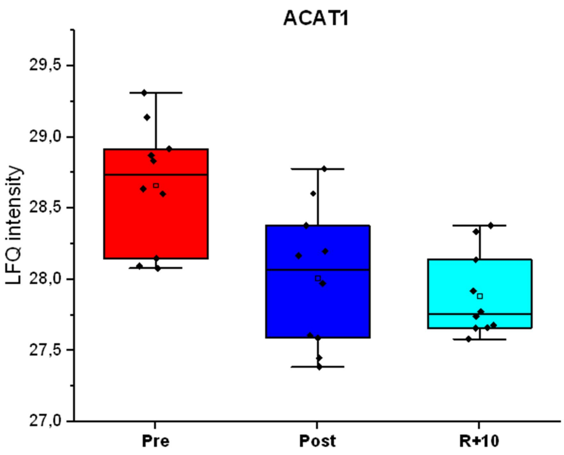
ACAT1
LFQ intensity
29,5
29,0
28,5
28,0
27,5
27,0
Pre
Post
R+10

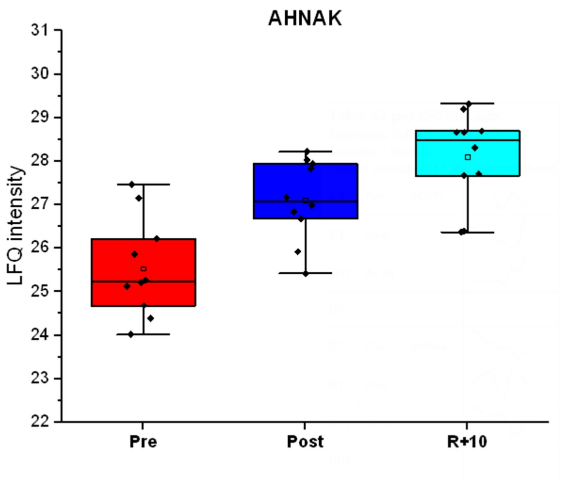
AHNAK
LFQ intensity
31
30
29
28
27
26
25
24
23
22
Pre
Post
R+10

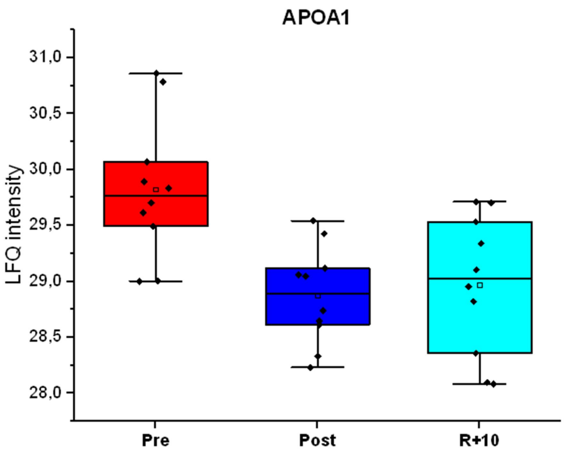
APOA1
LFQ intensity
31,0
30,5
30,0
29,5
29,0
28,5
28,0
Pre
Post
R+10

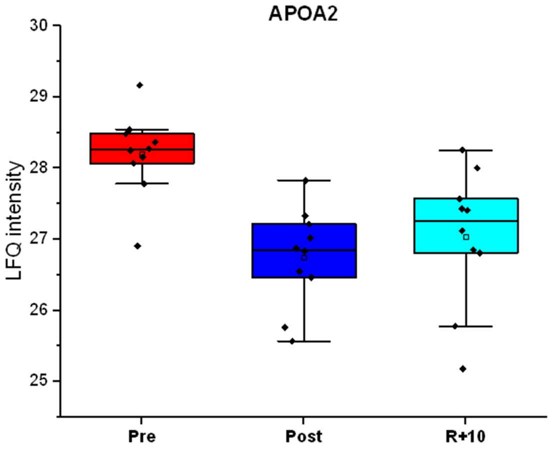
APOA2
LFQ intensity
30
29
28
27
26
25
Pre
Post
R+10

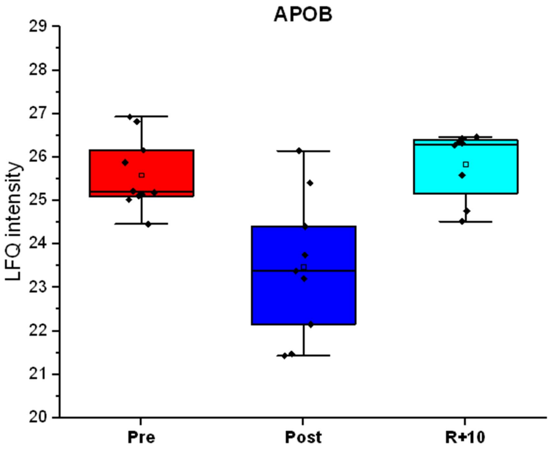
APOB
LFQ intensity
29
28
27
26
25
24
23
22
21
20
Pre
Post
R+10

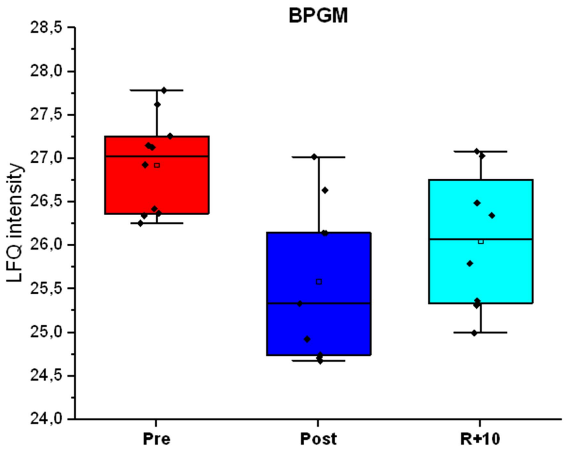
BPGM
LFQ intensity
28,5
28,0
27,5
27,0
26,5
26,0
25,5
25,0
24,5
24,0
Pre
Post
R+10

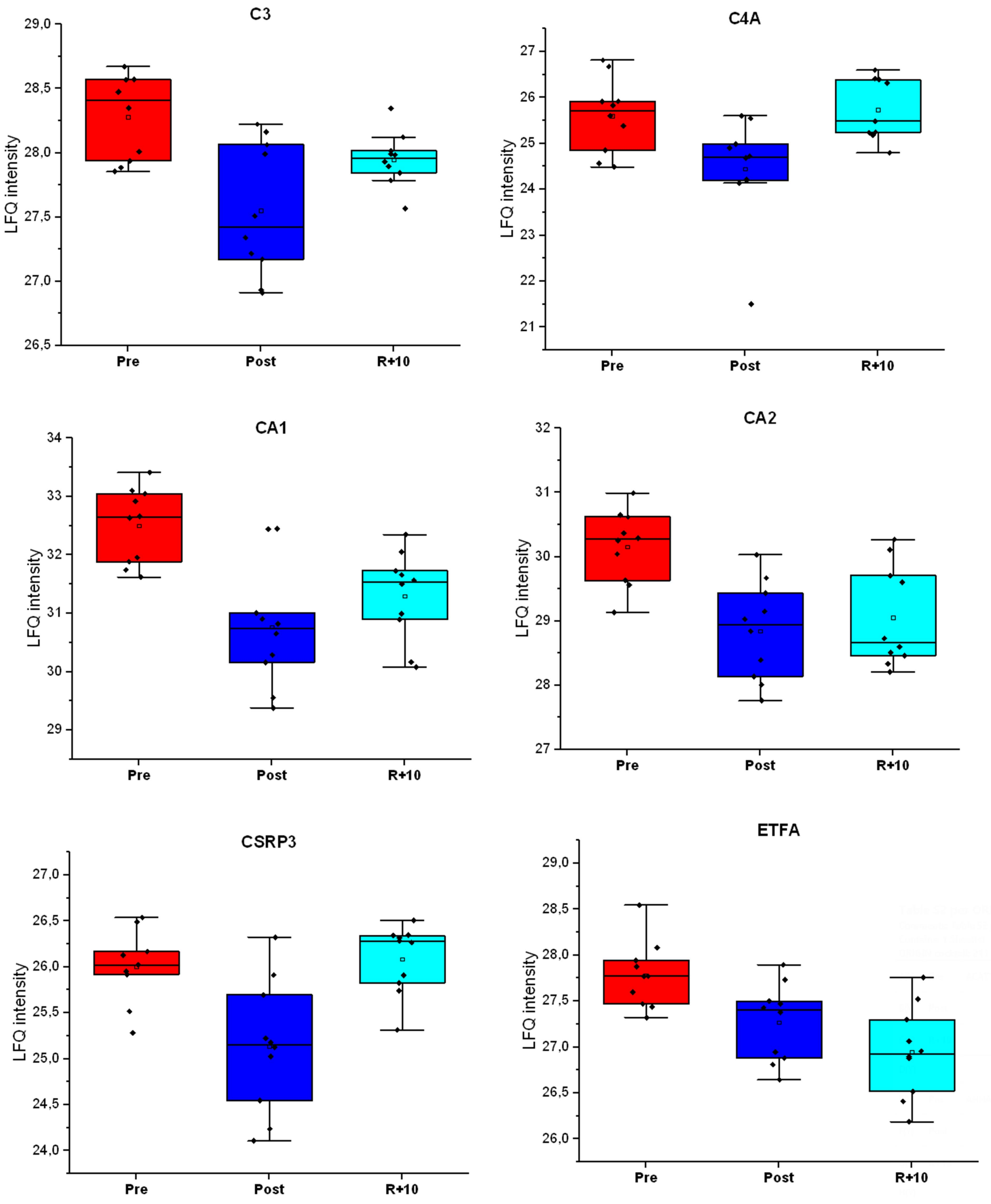
C3
LFQ intensity
29,0
28,5
28,0
27,5
27,0
26,5
Pre
Post
R+10
C4A
LFQ intensity
27
26
25
24
23
22
21
Pre
Post
R+10
CA1
LFQ intensity
34
33
32
31
30
29
Pre
Post
R+10
CA2
LFQ intensity
32
31
30
29
28
27
Pre
Post
R+10
CSRP3
LFQ intensity
27,0
26,5
26,0
25,5
25,0
24,5
24,0
Pre
Post
R+10
ETFA
LFQ intensity
29,0
28,5
28,0
27,5
27,0
26,5
26,0
Pre
Post
R+10

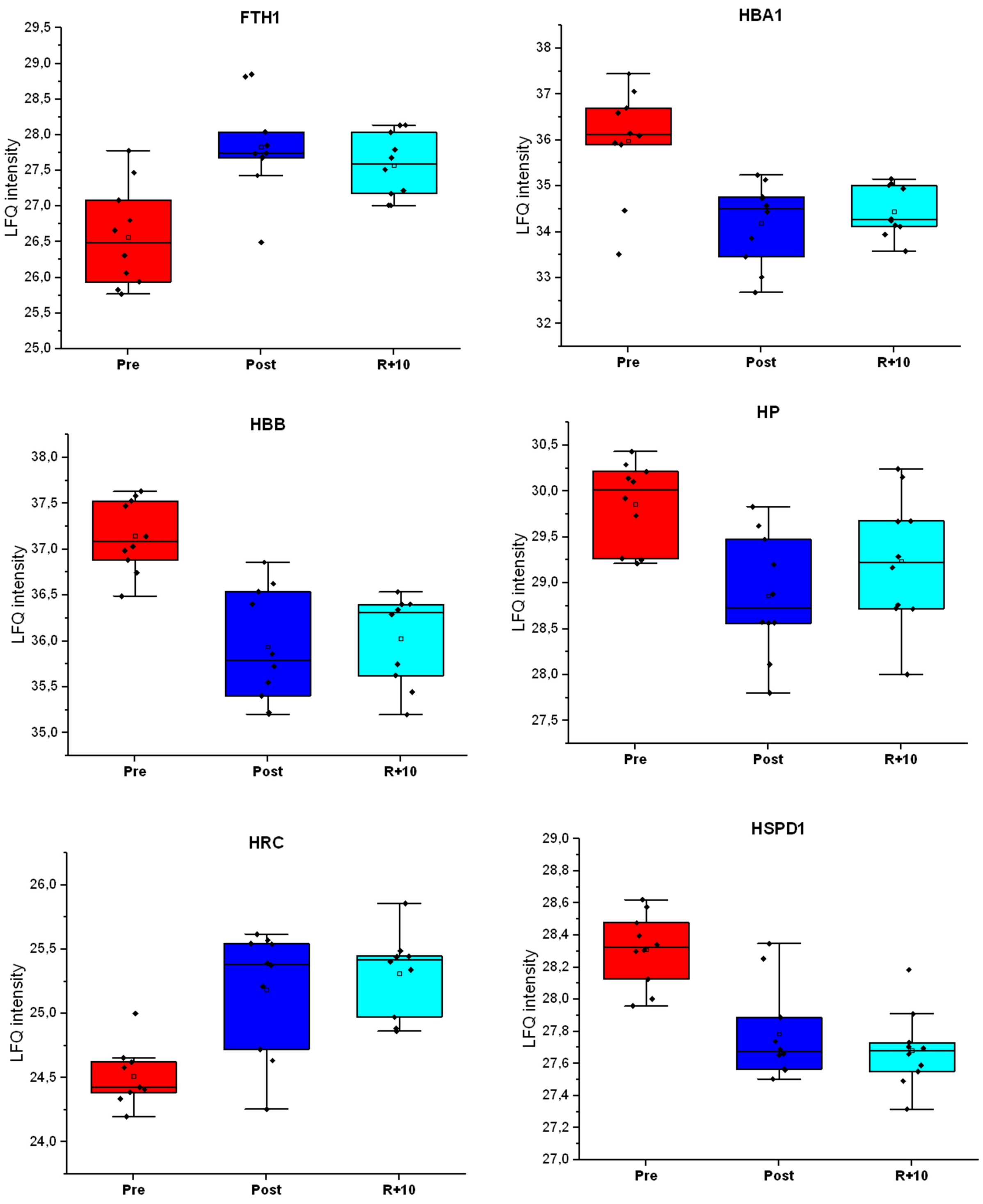
FTH1
HBA1
HBB
HP
HRC
HSPD1
LFQ intensity
Pre
Post
R+10

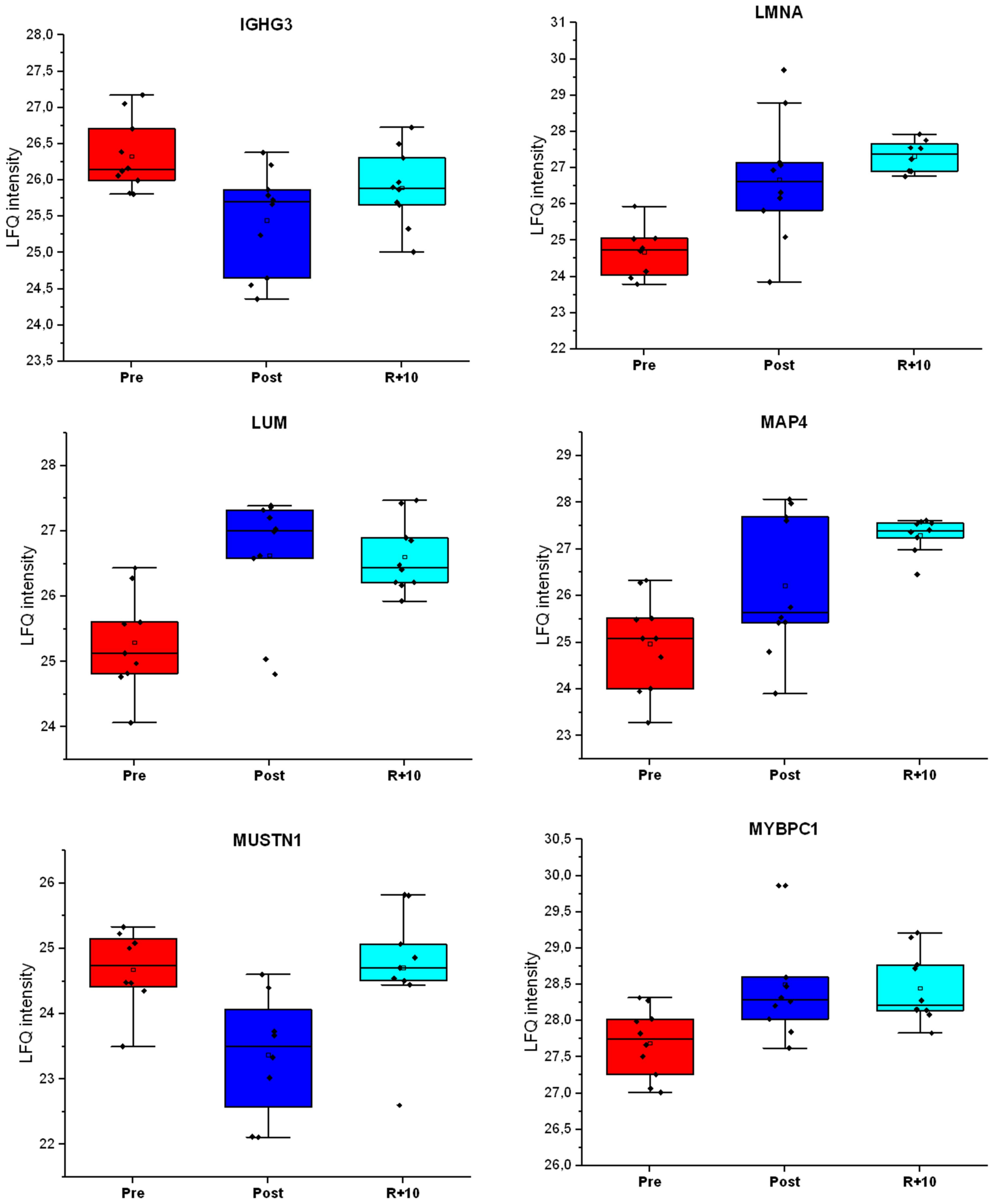
IGHG3
LFQ intensity
28,0
27,5
27,0
26,5
26,0
25,5
25,0
24,5
24,0
23,5
Pre
Post
R+10
LMNA
LFQ intensity
31
30
29
28
27
26
25
24
23
22
Pre
Post
R+10
LUM
LFQ intensity
28
27
26
25
24
Pre
Post
R+10
MAP4
LFQ intensity
29
28
27
26
25
24
23
Pre
Post
R+10
MUSTN1
LFQ intensity
26
25
24
23
22
Pre
Post
R+10
MYBPC1
LFQ intensity
30,5
30,0
29,5
29,0
28,5
28,0
27,5
27,0
26,5
26,0
Pre
Post
R+10

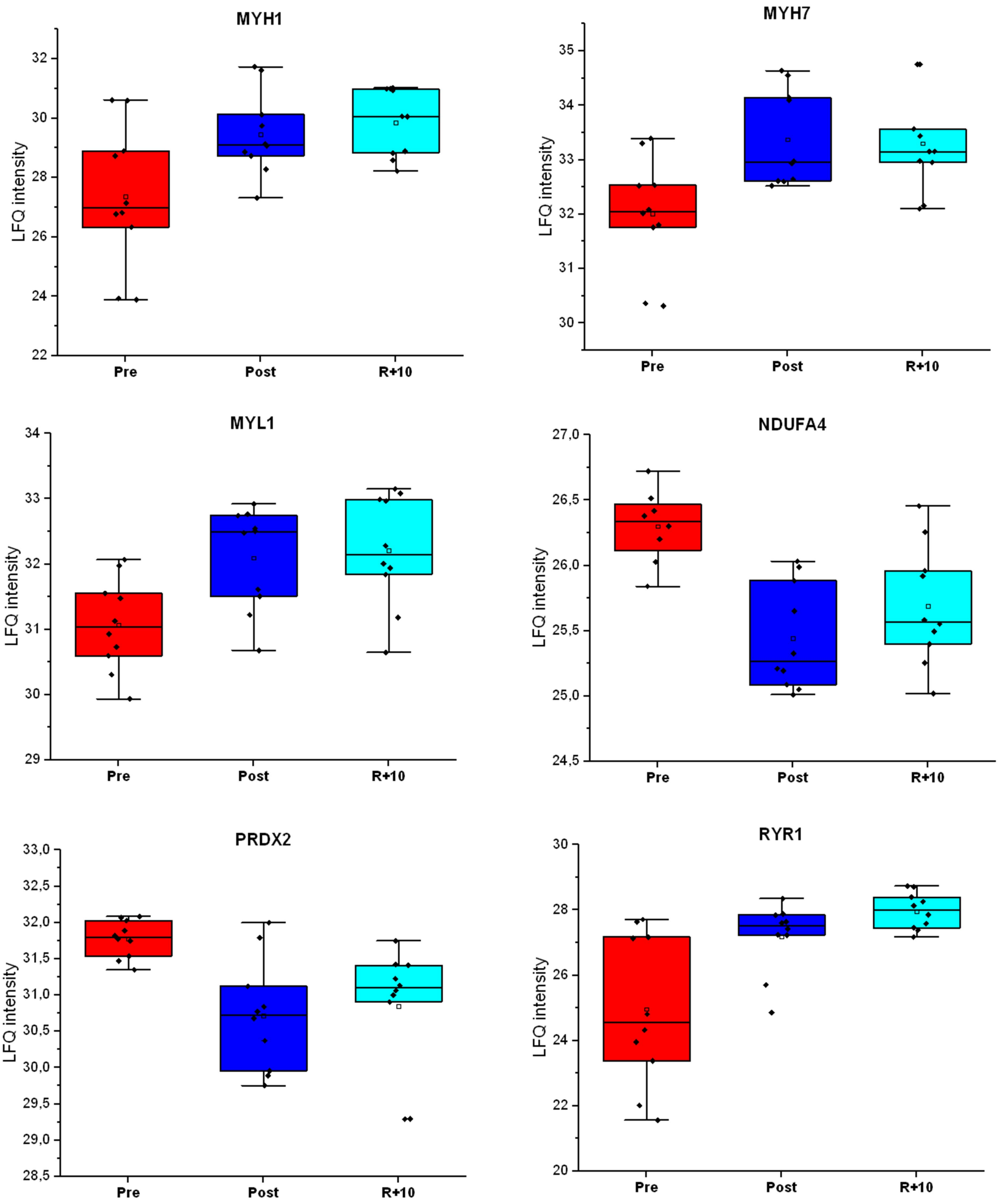

MYH1
LFQ intensity
22
24
26
28
30
32
Pre
Post
R+10
MYH7
LFQ intensity
30
31
32
33
34
35
Pre
Post
R+10
MYL1
LFQ intensity
29
30
31
32
33
34
Pre
Post
R+10
NDUFA4
LFQ intensity
24,5
25,0
25,5
26,0
26,5
27,0
Pre
Post
R+10
PRDX2
LFQ intensity
28,5
29,0
29,5
30,0
30,5
31,0
31,5
32,0
32,5
33,0
Pre
Post
R+10
RYR1
LFQ intensity
20
22
24
26
28
30
Pre
Post
R+10

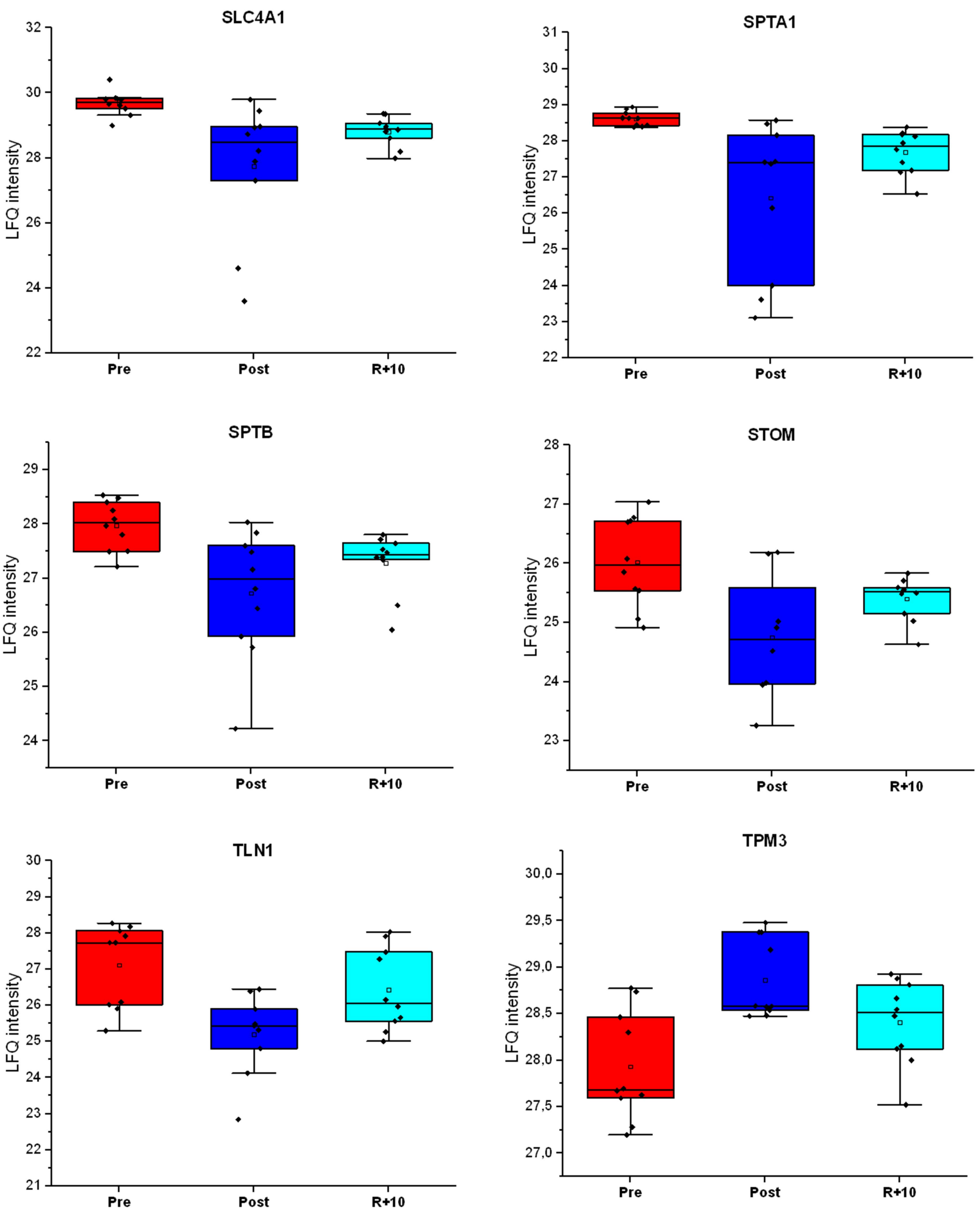
SLC4A1
LFQ intensity
32
30
28
26
24
22
Pre
Post
R+10
SPTA1
LFQ intensity
31
30
29
28
27
26
25
24
23
22
Pre
Post
R+10
SPTB
LFQ intensity
29
28
27
26
25
24
Pre
Post
R+10
STOM
LFQ intensity
28
27
26
25
24
23
Pre
Post
R+10
TLN1
LFQ intensity
30
29
28
27
26
25
24
23
22
21
Pre
Post
R+10
TPM3
LFQ intensity
30,0
29,5
29,0
28,5
28,0
27,5
27,0
Pre
Post
R+10

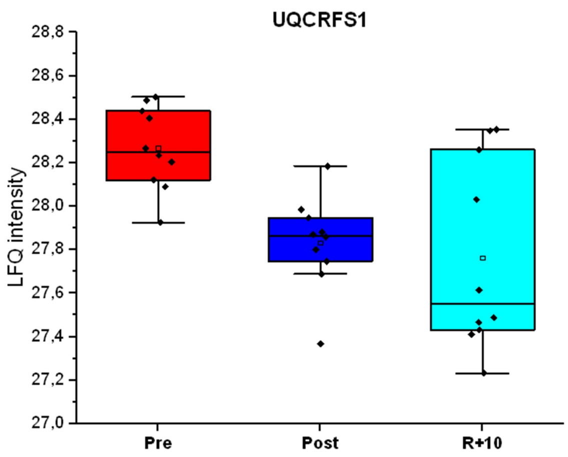


**Figure S2.** Box-plots of all differentially expressed proteins in Treatment bed rest subjects representing individual variability within the group (Pre, Post and R+10). LFQ = Label-free quantification.
