# Supplementary material for: Nitrosative Redox Homeostasis and Antioxidant Response Defense in Disused Vastus lateralis Muscle in Long-Term Bedrest (Toulouse Cocktail Study)
Source: Antioxidants (Basel). 2021 Mar 3;10(3):378. doi: 10.3390/antiox10030378 (PMC8001160; doi:10.3390/antiox10030378)
Supplement: Supplementary file 1 [file antioxidants-10-00378-s001.zip › antioxidants-1112224-supp-revise/Figure S3.pdf]

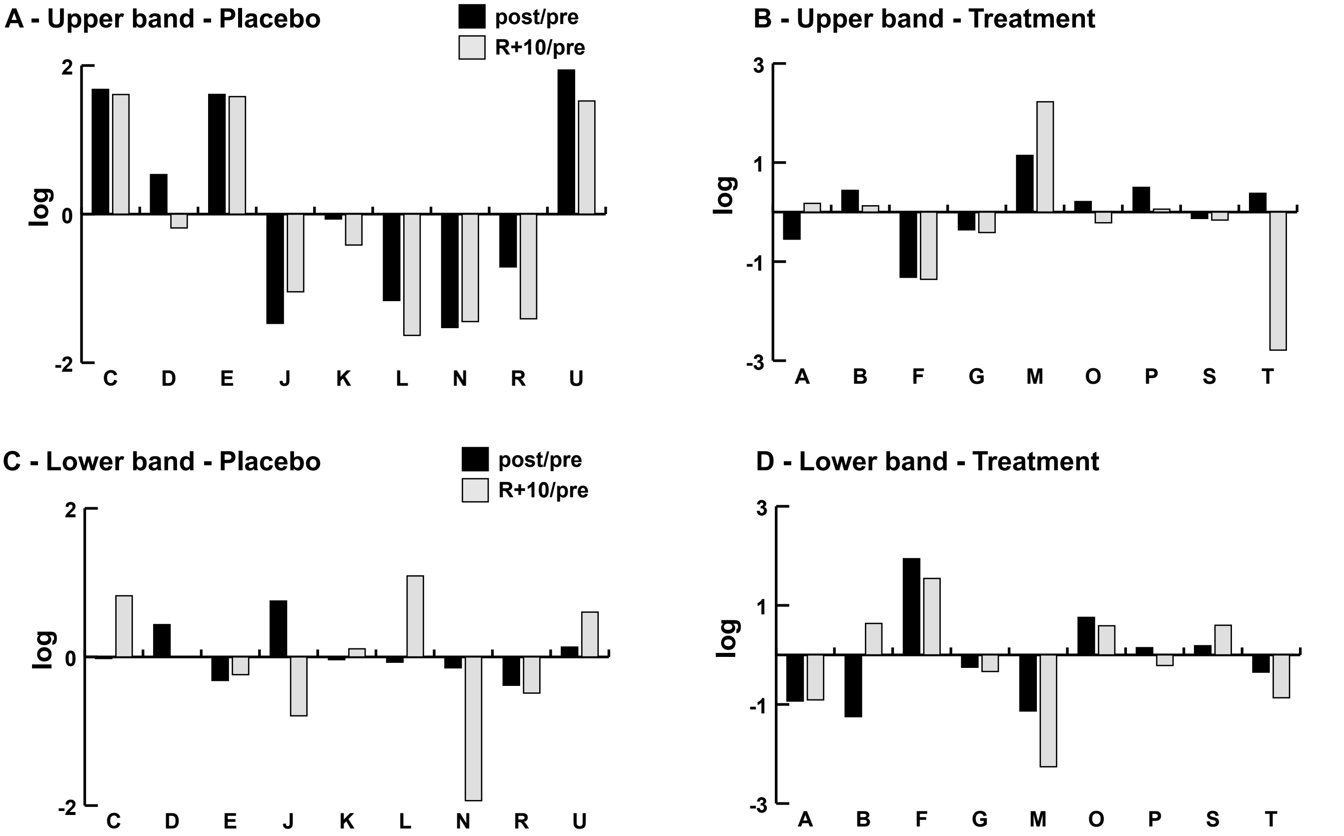

Figure S3. Tyrosine-Nitrated proteins detection. ECL detection in monodimensional acrylamide gel 12% T 4% C, followed by immunobloting with Monoclonal Anti 3-nitrotyrosine Clone 18G4 (Sigma-Aldrich). Results normalized with the total protein binding-dye for membrane SYPRO Ruby. Two main bands are detected with the antibody. (A) and (B) Graphics of Placebo and Treatment subjects upper band. (C) and (D) Graphics of Placebo and Treatment subjects lower band. Normalization on pre-bed rest values.
