# Supplementary material for: Nitrosative Redox Homeostasis and Antioxidant Response Defense in Disused Vastus lateralis Muscle in Long-Term Bedrest (Toulouse Cocktail Study)
Source: Antioxidants (Basel). 2021 Mar 3;10(3):378. doi: 10.3390/antiox10030378 (PMC8001160; doi:10.3390/antiox10030378)
Supplement: Supplementary file 1 [file antioxidants-10-00378-s001.zip › antioxidants-1112224-supp-revise/Figures S4.pdf]

Method. *Nuclear factor-erythroid-2-related factor 2 (Nrf-2) myonuclear localization*

A detailed subcellular localizzation analysis of Nrf2 in myonuclei indirectly is an index of the transcription factor activation ratio.

Human skeletal muscle biopsy cryosections (8 μm thickness), from *VL* (n=7) pre-, post- and rec-BR groups, were double immunolabelled with anti-Dystrophin (mouse monoclonal 107416, Novocastra, Leica Biosystems.com) and anti-Nrf-2 (rabbit polyclonal, sc-13032, Santa Cruz, Santa Cruz Biotechnology, Inc.) antibodies. In all Nrf-2 immunostaining protocols goat anti-rabbit Alexa-488 for Nrf-2 and goat anti-mouse Alexa-555 for Dystrophin were used. Nuclei were routinely counterstained with blue DAPI.

Nrf-2 double immunostained muscle cryosections were inspected by confocal microscopy (Leica TCS SP8, leica-microsystems.com) for high resolution immunosignal detection of Nfr2-accumulation in myonuclei. At least 70 myonuclei were scored for each pre-, post-BR, and R+10 muscle biopsy/subject.

Figure S 2. Anti-oxidative master gene Nrf-2 regulation in *VL* of bed rest subjects. **a**. Representative image Nrf-2 immunopositive myonuclei (green) in *VL* before (Pre) and after (Post) bedrest in CTR and Cocktail group. Dystrophin (red) used as reference marker to label the subsarcolemmal plasma membrane border; nuclei were counterstained with DAPI (blue). Inset, magnification Nrf-2 positive myonuclei for comparison with Nrf-2 negative myonuclei and nuclei outside the myofiber. **b.** Quantitative analysis of Nrf-2 positive myonuclei in *VL*. The total number of Nrf-2 positive myonuclei was decreased in both *VL* CTR (Pre, 18.95 $\pm$ 3.56%, Post, 2.38 $\pm$ 0.94%, $p \leq 0.001$) and Cocktail (Pre, 23.4 $\pm$ 4.43%, Post, 8.27 $\pm$ 1.94%, $p \leq 0.05$) group. (* = significant difference, ANOVA and *t-test*, n= 10, $p\text{-value} \leq 0.05$). The graph represents the mean ± SEM.

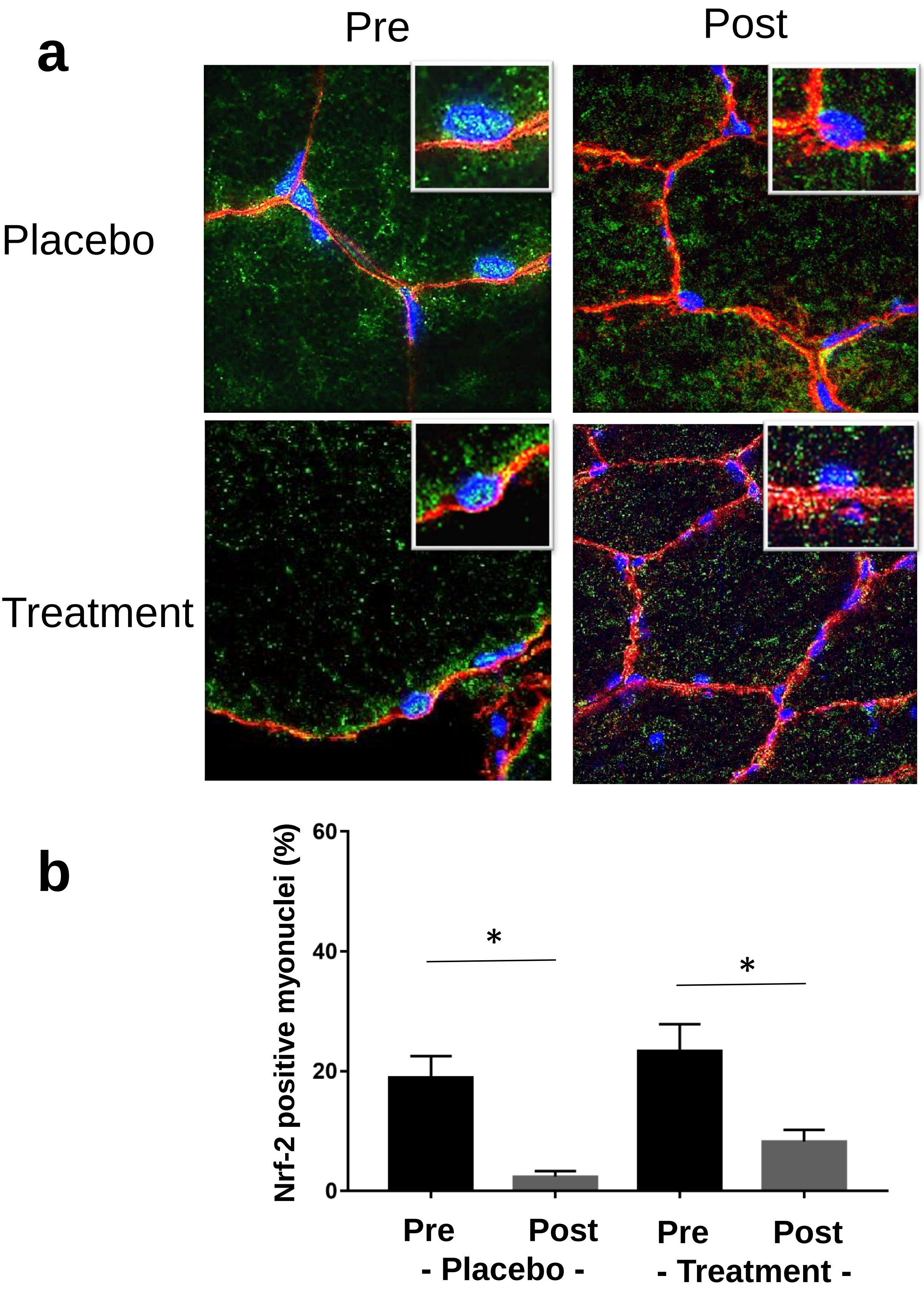
a
Pre
Post
Placebo
Treatment
b
Nrf-2 positive myonuclei (%)
60
40
20
0
*
*
Pre
Post
Pre
Post
- Placebo -
- Treatment -

Figure S4
